# Transcriptional Reprogramming in Prostate Cancer: Altered AR Activity, the Modulatory Role of CDK8/19, and Prospects for Combination Therapy

**DOI:** 10.3390/ijms27219855

**Published:** 2026-11-03

**Authors:** Maria Dodina, Polina Pavlova, Polina Kuzkokova, Aleksey Krasnyi, Alexander Shtil, Roman Ivanov, Ekaterina Minskaia

**Affiliations:** 1 Translational Medicine Research Center, Sirius University of Science and Technology, 1 Olympic Avenue, 354340 Sochi, Russia; 2 Institute of Experimental Oncology and Carcinogenesis, Blokhin National Medical Research Center of Oncology, 24 Kashirskoye Shosse, 115522 Moscow, Russia

**Keywords:** prostate cancer, androgen receptor, cyclin-dependent kinases, castration resistance, lineage plasticity

## Abstract

Prostate cancer (PCa) remains a leading cause of cancer-related mortality in men, with resistance to androgen-deprivation therapy (ADT) being the major clinical challenge. A key driver of this resistance is transcriptional reprogramming, during which androgen receptor (AR) activity shifts from ligand-dependent signaling to reactivation via splice variants such as AR-V7 and eventually to AR-negative states. The oncogene c-MYC plays a pivotal role through context-dependent crosstalk with AR, suppressing canonical AR-dependent transcription while promoting its own oncogenic program. This reprogramming is sustained by dysregulation of RB1/E2F, NF-κB, PI3K/AKT/mTOR, WNT/β-catenin, and Hippo/YAP/TAZ pathways, alongside N-MYC and L-MYC activation, forming an interconnected network driving phenotypic plasticity. This review focuses on cyclin-dependent kinases 8 and 19 (CDK8/19), components of the Mediator complex kinase module, as potential regulators of epigenetic plasticity in PCa capable of modulating both AR-dependent and AR-independent transcription. Preclinical studies show that CDK8/19 inhibition suppresses castration-resistant prostate cancer (CRPC) xenograft growth, restores ADT sensitivity, and additively enhances enzalutamide even in AR-V7-expressing resistant models, though the mechanisms of CDK8/19-mediated AR regulation remain largely undefined. We analyze the signaling pathways driving AR activity changes during PCa progression, evaluate potential mechanisms of CDK8/19-mediated AR regulation, and assess the therapeutic potential of CDK8/19 inhibitors with ADT.

## 1. Introduction

Prostate cancer (PCa) remains one of the most common malignancies and a leading cause of cancer-related death among men worldwide [1]. The cornerstone of systemic treatment for advanced PCa has traditionally been androgen-deprivation therapy (ADT), aimed at reducing circulating androgen levels or blocking the androgen receptor (AR) signaling pathway [2]. The introduction into clinical practice of potent next-generation AR signaling inhibitors (enzalutamide and abiraterone) has improved patient survival. However, despite high initial efficacy, a significant proportion of patients develop castration-resistant prostate cancer (CRPC), which remains a major cause of poor outcomes in advanced PCa.

One of the key mechanisms driving CRPC formation is the reactivation of AR signaling—through amplification and overexpression of the *AR* gene, the emergence of constitutively active splice variants (such as AR-V7), and other mechanisms [3,4]. Further disease evolution may be accompanied by the development of various resistant phenotypes, among which one of the most aggressive is neuroendocrine prostate cancer (NEPC), characterized by the loss of AR dependence [5]. However, such disease evolution is not necessarily linear: AR-dependent, AR-low, and neuroendocrine phenotypes may represent distinct or overlapping states of castration resistance. Thus, in PCa, therapeutic pressure reveals a systemic issue—lineage plasticity [6]. This process demonstrates that therapies targeting exclusively the ligand-binding domain of the receptor have fundamental conceptual limitations: they do not deprive the tumor of its ability to undergo reprogramming.

This review focuses on the concept of transcriptional vulnerability in adapting tumor cells. In the context of PCa, this reprogramming involves both the reactivation of AR signaling under reduced androgen levels and the abandonment of AR dependence. A large-scale change in cellular phenotype is impossible without the involvement of master regulators that link activated compensatory survival pathways to the basal transcriptional machinery. One likely regulator of epigenetic plasticity in PCa is the Mediator complex module containing the paralogs cyclin-dependent kinases 8 and/or 19 (CDK8/19) [7,8,9]. Accumulating evidence indicates that CDK8/19 can modulate AR-dependent transcriptional programs, positioning it as a key factor in transcriptional reprogramming in CRPC. Specifically, CDK8/19 inhibition suppresses the growth of CRPC xenografts, restores sensitivity to ADT [10], and enhances the effect of enzalutamide, including in resistant cell lines expressing AR-V7 [11].

The aim of this review is to systematize current knowledge on the dynamics of AR signaling at various stages of PCa progression, integrate insights into the modulatory role of CDK8/19 in this process, and, based on a comprehensive analysis, discuss potential combination therapy strategies aimed at overcoming resistance and blocking tumor plasticity. To achieve this goal, the review will examine the versatility of AR: from the classical transcriptional program to the development of ligand-independent activation (including via AR-V7) and subsequent attenuation of AR signaling; it will also describe the mechanisms of CDK8/19 function within the kinase module of the Mediator, their involvement in transcriptional regulation, interaction with chromatin-remodeling complexes, and possible role in adaptive transcriptional reprogramming in PCa, substantiating its role as an AR coactivator. To ensure clarity and distinguish between established facts and theoretical models, we classified the discussed molecular mechanisms into three categories: (1) mechanisms validated directly in prostate cancer models (by default, unless otherwise indicated); (2) mechanisms established in non-PCa models (N-PCa), including other malignancies, non-malignant cell lines, and model organisms, but not yet fully characterized in PCa; and (3) mechanistic extrapolations/hypotheses (HYP), representing logical inferences lacking direct experimental validation.

In light of these data, a critical evaluation of existing therapeutic strategies will be conducted, and novel combination approaches aimed at modulating both the tumor itself and its microenvironment will be considered.

## 2. The Versatile Role of AR in Prostate Cancer Progression

### 2.1. AR Functions in Prostate Cells

#### 2.1.1. AR Structure

The androgen receptor is a ligand-dependent transcription factor (TF) of the steroid nuclear receptor family, which serves as the primary driver of PCa progression. By binding androgens (such as testosterone and dihydrotestosterone), AR activates the expression of target genes and thereby regulates cell proliferation, differentiation, and survival.

The AR protein consists of 919 amino acid residues and comprises four domains. The N-terminal domain (NTD) is a large, predominantly disordered region containing the transcriptionally active activation function-1 (AF-1) and an FxxLF motif, which interacts with the C-terminal domain of the receptor and is critically important for the regulation of target gene expression [12]. The DNA-binding domain (DBD) is a compact globular domain with two zinc fingers; it recognizes androgen-response elements (AREs) within target genes and facilitates AR dimerization on DNA, thereby conferring gene-specificity to the transcriptional response [13]. The ligand-binding domain (LBD) is located at the C-terminus and is responsible for androgen binding [14]. Unlike other nuclear receptors, upon ligand binding, AR does not form a classical AF-2 pocket that distinguishes the specific LxxLL coregulator motif. Instead, the AR AF-2 forms a shallow hydrophobic pocket that recognizes the FxxLF motif at the receptor’s N-terminus or on coregulators [15]. The DBD and LBD are connected by a short flexible linker—the hinge region (HD)—which contains a nuclear localization signal (NLS) and also participates in coregulator binding [16].

#### 2.1.2. Mechanism of AR Action

In the absence of a ligand, the receptor resides in the cytoplasm in an inactive form, bound to chaperones, particularly Hsp70 and Hsp90, which maintain the protein in a conformation favorable for ligand binding [17]. Upon androgen binding, AR undergoes conformational changes, resulting in its dissociation from these chaperones and translocation to the nucleus as monomers, where it dimerizes via the D-box within the DBD, forming head-to-head and tail-to-tail contacts [18]. Additional contacts between the NTD, DBD, and LBD form a single dimer in which conformational changes in one domain (e.g., upon ligand or coregulator binding) are transmitted to the dimer partner and alter the transcriptional activity of the complex.

ARE is a palindromic DNA sequence consisting of two hexameric half-sites [19]. Each half-site is recognized by one AR monomer via one of the two zinc fingers of the DBD, while the second finger of each monomer mediates their interaction, forming an AR homodimer on DNA [20]. In addition to full-length palindromic motifs, AR is also capable of binding to half-site AREs (hAREs) containing only a single hexameric motif [21]. In vitro studies have shown that such sites can associate with monomeric AR [22], and footprinting data also indicate that AREs may be occupied by a single AR molecule [23]. Chromatin ImmunoPrecipitation sequencing (ChIP-seq) studies in PCa models show that as the disease progresses, the proportion of half-site motifs among AR sites increases, with many of these hAREs localized in enhancers and intergenic regions and located adjacent to motifs of other factors that interact with AR, such as FOXA1 and HOXB13, as well as E2F, MYC/MAX, and the STAT family [24,25]. This supports the concept that the monomeric form of AR at half-sites and composite motifs may play a significant role in AR cistrome reprogramming and the activation of pro-oncogenic transcriptional programs during PCa progression. Nevertheless, the mere presence of a motif is insufficient for AR binding to DNA: in most chromatin regions containing AREs or hAREs, AR is not detected on DNA [26]. This points to the existence of an additional level of regulation in the selection of AR DNA-binding sites, linked to the recruitment of TFs and coregulators that determine which potential AREs/hAREs become functional in a given context [27,28].

The TFs FOXA1, HOXB13, and GATA2 are important regulators of AR cistrome formation and have traditionally been viewed as pioneer factors in PCa [29,30]. However, recent studies indicate that there is a clear hierarchy among these factors: only FOXA1 possesses the ability to recruit AR to binding sites independently of the H3K4me1 histone mark, subsequently attracting HOXB13 and GATA2 to enhance factor interaction on chromatin [27]. Notably, HOXB13 also interacts with SMARCD2, a member of the SWItch/Sucrose Non-Fermentable (SWI/SNF) chromatin-remodeling complex, altering chromatin accessibility in PCa [31]. In addition, AR activity is modulated by coregulators of the SRC-1/2/3 family, histone acetyltransferases p300/CBP, and the Mediator complex (notably MED1), as well as by the SWI/SNF remodeling complexes and the epigenetic enzymes LSD1 and EZH2, which possess context-dependent functions and, depending on the composition of the regulatory complex and chromatin environment, can promote either activation or repression of AR-dependent genes [22,32,33]. In contrast, the corepressors NCoR and SMRT, along with deacetylases HDAC and SIRT1, can attenuate AR-dependent transcription, and the imbalance between coactivator and corepressor circuits is considered one of the key mechanisms underlying AR signaling reprogramming during PCa progression [22,25].

#### 2.1.3. Canonical AR Transcriptional Program

In the intact prostate gland, AR regulates genes responsible for epithelial differentiation and prostate-specific metabolism, including secretory function [34].

Among the best-known AR target genes is the kallikrein family *KLK3* and *KLK2*, which are classical markers of differentiation and secretory activity of the prostate epithelium [34]. The *KLK3* gene encodes the prostate-specific antigen (PSA), which serves as a key biomarker for monitoring PCa progression and assessing treatment response [35]. Another important androgen-regulated gene is *TMPRSS2*, encoding a type II transmembrane serine protease. In the normal gland, the role of TMPRSS2 is not fully defined, but it is thought to participate in maintaining prostate homeostasis and male fertility through proteolytic activation of endogenous substrates such as ENaC, PAR-2, and KLK2 [36]. Expression of the *FKBP5* gene is also positively regulated by AR, and FKBP5 stabilizes AR and sustains its signaling, acting as a co-chaperone in complex with Hsp90 [37,38].

The *NKX3.1* gene is also a direct transcriptional target of AR; together with AR, NKX3.1 regulates the transcription of secretory protein genes, including *KLK3*. NKX3.1 is known to possess pronounced anti-oncogenic properties: it promotes p53 stabilization and suppresses the PI3K/AKT pathway, thereby limiting the proliferation and invasiveness of prostate cells [39,40]. Interestingly, NKX3.1 also plays a role in regulating stem cell properties. It has been detected in a rare population of castration-resistant cells expressing NKX3.1 (CARNs), which possess stem cell properties during prostate regeneration. Furthermore, its role in activating *OCT4* expression has been demonstrated, allowing its use as a substitute for OCT4 in generating induced pluripotent stem cells [40].

Collectively, genome-wide ChIP-seq and transcriptomic data indicate that AR in the prostate binds to numerous regulatory regions and mediates androgen-dependent regulation of multiple genes, with a notable proportion of these targets undergoing not only activation but also repression [41,42].

### 2.2. AR Activity During PCa Progression

#### 2.2.1. Changes in the AR Transcriptional Program

In PCa, dependence on context-dependent transcription is already-established at the primary tumor stage. During progression to CRPC and subsequently to metastatic CRPC (mCRPC), the AR cistrome undergoes large-scale reprogramming: a significant proportion of normal AR binding sites cease to function, and oncogene-specific sites become activated. A systematic analysis of AR binding sites at different stages of PCa progression by Severson et al. identified four gene sets: LiT (186 genes whose expression is lost in tumors), GiT (159 genes whose expression is gained in tumors), LiM (156 genes whose expression is lost in metastases), and GiM (267 genes whose expression is gained in metastases). Genes of the LiT program, associated with normal differentiation functions, when downregulated predict shorter metastasis-free survival in a cohort of patients with a high rate of PCa recurrence, whereas the GiM program is enriched in cell-cycle and DNA replication genes, and its high activity is associated with poor survival in mCRPC [43]. Collectively, these data indicate that AR cistrome reprogramming serves as a switch from differentiation-oriented to proliferative and metastatic transcriptional programs.

Pioneer factors, discussed in the previous section, play a significant role in AR cistrome reprogramming. It has been shown that FOXA1 and HOXB13 colocalize with reprogrammed AR binding sites, and overexpression of both factors in the immortalized prostate cell line LHSAR was sufficient to recapitulate the cistrome state characteristic of PCa tumors [44]. Moreover, it has been demonstrated that in NEPC, a cardinal epigenetic rearrangement occurs: the promoters of *AR*, *GATA2*, and *HOXB13* become hypermethylated, FOXA1 protein becomes undetectable in ~90% of NEPC samples, and *AR* mRNA levels drop to nearly zero [27].

An additional layer of transcriptional reprogramming is provided by oncogenic factors of the ETS family. In primary PCa, they are frequently activated due to structural genomic rearrangements that result in the fusion of the ETS-factor coding region with regulatory elements of an actively expressed gene. The most characteristic example is the *TMPRSS2-ERG* rearrangement, in which *ERG* comes under the control of the androgen-regulated *TMPRSS2* promoter, leading to its pronounced AR-dependent expression. Similar, though considerably rarer, rearrangements involve *ETV1*, *ETV4*, and *FLI1*, with various genes harboring active promoters or enhancers acting as their partners. In the TCGA primary PCa cohort, ETS rearrangements were detected in approximately 59% of tumors: *ERG* in about 46%, *ETV1* in 8%, *ETV4* in 4%, and *FLI1* in about 1% [45]. The *TMPRSS2-ERG* rearrangement is accompanied by ERG overexpression, which substantially affects the AR cistrome. ERG directly interacts with AR via an AR-interacting motif within the ETS domain and can enhance AR binding to DNA [46].

Thus, AR cistrome reprogramming is not a secondary phenomenon but a central molecular event that determines the potential for PCa progression.

#### 2.2.2. Mechanisms of CRPC Development

In the transition from hormone-sensitive PCa to CRPC, AR retains or restores transcriptional activity despite low (castration-level) androgen concentrations. The mechanisms underlying this transition are conventionally divided into AR-dependent—including genomic aberrations of the receptor itself and its activation in a ligand-independent manner—and AR-independent, the main representatives of which are activation of alternative nuclear receptors and the phenomenon of lineage plasticity.

Among AR-dependent mechanisms, amplification of the *AR* gene occupies a central position: increased copy numbers are detected in 30–50% of mCRPC cases, and in patients progressing on enzalutamide therapy this figure reaches 53% [3,47]. At the same time, 15–26% of patients exhibit high *AR* mRNA levels in the absence of gene amplification; in such cases, amplification of upstream *AR* enhancers plays a key role. In a representative cohort of 101 CRPC samples, co-amplification of the *AR* gene and the upstream enhancer (~650 kb upstream of the transcription start site) was detected in 67% of cases, whereas isolated enhancer amplification occurred in 13%. In addition to the upstream enhancer, a downstream enhancer (~65 kb downstream of the *AR* gene termination site) as well as intragenic enhancers in introns 2 and 3 have been described; these become activated due to dysregulation of EZH2, ROR-γ, and SMAD3 under ADT [48].

AR point mutations constitute an additional AR-dependent mechanism of resistance, and their frequency varies according to disease stage, prior therapy, biospecimen type, and the analytical method used. In a clinicogenomic cohort of 540 patients with CRPC, AR point mutations were detected in 8.2% of patients before AR-directed therapy and in 18.5% of patients after treatment. L702H was the most frequent mutation detected after therapy, occurring in 9.9% of patients [49]. Most clinically relevant mutations are located in the LBD. L702H can permit glucocorticoid-mediated activation of AR, whereas H875Y and T878A broaden AR ligand specificity. T878A is associated with resistance to abiraterone and can convert hydroxyflutamide and nilutamide from AR antagonists into AR agonists [50,51].

The F877L mutation, referred to as F876L in earlier studies, can convert enzalutamide and apalutamide (ARN-509) from AR antagonists into AR agonists in cell-based and xenograft models [52,53]. However, F877L is not a common alteration in mCRPC. In a phase I study of ARN-509, F876L-encoding mutations were detected by BEAMing in circulating tumor DNA (ctDNA) from three of 29 patients during treatment but were not detected in pretreatment samples [52]. In a subsequent apalutamide study, F877L was detected at baseline in two of 93 patients and was newly detected during treatment in three of 82 patients; all three post-treatment detections occurred in patients with mCRPC [54]. A practical consequence of LBD agonist-switching mutations is the Antiandrogen Withdrawal Syndrome (AAWS), sometimes observed after discontinuation of first-generation antiandrogen therapy, when instead of the expected tumor growth due to blockade relief, patients experience a temporary decline in PSA levels and even tumor shrinkage [55]. It should also be noted that NTD mutations account for about one-third of all AR mutations and enhance the receptor’s transcriptional activity, increase its stability, and promote coregulator recruitment [3].

It is also noteworthy that certain AR polymorphisms initially make some individuals more sensitive to androgens, which may facilitate the development of castration resistance in PCa. For example, the length of the polymorphic CAG repeat region encoding the polyglutamine tract in the NBD of AR, as a rule, inversely correlates with AR transcriptional activity [56]. Epidemiological observations show that short CAG repeats are associated with higher AR transactivation activity in vitro, and in African Americans they are more often associated with increased tumor aggressiveness, which may partly explain racial differences in PCa incidence [57]. At the same time, some studies have not found a direct association between CAG repeat length and PCa risk within this racial group, which points to the complex and multifactorial nature of the observed differences [58].

In addition to genomic alterations, AR can be activated in the absence of a ligand through paracrine and cellular signaling pathways. In particular, IL-6 induces transcription of canonical AR targets via STAT3, which is active in 95% of mCRPC cases [59,60]. Notably, activation of the NF-κB pathway and, consequently, IL-6/STAT3 signaling promotes AR-V7 overexpression [61,62]. Ligand-independent activation mechanisms of AR also involve the kinases AKT and HER2: AKT can directly phosphorylate AR, whereas HER2 acts through activation of downstream kinase cascades [63,64,65]. Furthermore, upon activation of the WNT/β-catenin pathway, stabilized β-catenin directly interacts with AR, enhancing its transcriptional activity [66]. The influence of activation of these and other pathways on AR transcriptional activity will be discussed in more detail in subsequent sections.

AR-independent resistance mechanisms primarily include activation of the glucocorticoid receptor (GR). Normally, AR suppresses GR transcription by binding to intronic regulatory elements of the *GR* gene, but under ADT this suppression is relieved, leading to more than a twofold increase in GR expression. Because a portion of AR binding sites overlap with GR sites, GR is able to activate AR-dependent programs bypassing AR blockade [66]. Another fundamentally important mechanism is epigenetically mediated transdifferentiation into NEPC, which occurs in approximately 20% of CRPC cases. The key genetic drivers of this plasticity are concurrent loss of *TP53* and *RB1*, which activates EZH2-mediated silencing of *AR* and luminal genes, and *SOX2*, which triggers a JAK-STAT-dependent transition to a multipotent stem-cell-like state. Neuroendocrine transition is characterized by activation of the TFs ASCL1, FOXA2, and NKX2-1, as well as expression of the markers INSM1, SYP, and NCAM1 [66]. Collectively, the mechanisms listed above reflect the biological plasticity of CRPC, which necessitates the development of therapeutic strategies targeting multiple resistance mechanisms simultaneously.

##### Special Role of Truncated AR Variants

At present, more than 20 different androgen receptor variants (AR-Vs) are known, most of which lack a functional ligand-binding domain and are consequently constitutively active in the absence of androgens [4]. Among these, AR-V7 (also known as AR3) is one of the most common and ubiquitously expressed isoforms in PCa and demonstrates the strongest association with resistance to drugs aimed at reducing AR transcriptional activity, as well as with poor prognosis. From a clinical perspective, the frequency of AR-V7 detection depends substantially on disease stage, prior treatment exposure, biospecimen type, and the detection method; therefore, it should not be presented as a single estimate applicable to all patients with PCa or mCRPC. In the tissue-based study by Sharp et al., nuclear AR-V7 protein expression, assessed by immunohistochemistry using the RM7 antibody, was detected in one of 358 primary PCa specimens. In the same study, detectable nuclear AR-V7 expression was observed in 15 of 20 biopsies obtained after progression on primary androgen-deprivation therapy, with or without bicalutamide, and before the initiation of standard systemic therapy for CRPC [67]. Different estimates have been reported in circulating tumor cell (CTC)-based studies in mCRPC: *AR-V7* mRNA was detected by reverse-transcription PCR in 39% of patients before initiation of enzalutamide and in 19% of patients before initiation of abiraterone, in two cohorts of 31 patients each [68]. In the PROPHECY study, AR-V7 was detected in 28 of 116 patients (24%) by CTC *AR-V7* mRNA analysis and in 11 of 107 patients (10%) by assessment of nuclear AR-V7 protein in CTCs [69]. As a result of alternative splicing, AR-V7 retains intact NTD and DBD, a partially truncated HD, but lacks LBD, which is replaced by a short peptide sequence of 16 amino acids encoded by cryptic exon 3 (CE3) [70,71]. Nuclear import of AR-V7 is independent of the canonical NLS-mediated pathway involving importin-α/β, exhibits significantly faster kinetics compared with full-length AR (AR-FL), and presumably employs the D-box within the DBD as a nuclear transport element [72]. Notably, AR-V7 possesses high intranuclear mobility and interacts with chromatin transiently with an extremely low binding occupancy (~0.01% of input signal by ChIP-qPCR, whereas for ligand-activated AR-FL this value is 6–12%), which, in combination with high transcriptional activity, points to the implementation of a “hit-and-run” model—whereby a TF transiently binds to DNA, recruits stable cofactors, and leaves the site [72,73]. It should be emphasized that the use of this model by repressive TFs significantly complicates the detection of their involvement in transcriptional regulation by standard methods, including ChIP-seq [74]. At the same time, according to some data, AR-V7 indeed possesses increased repressive function compared with AR-FL: reliably downregulated genes include *B4GALT1*, encoding galactosyltransferase involved in post-translational glycosylation of PD-L1; *SLC30A7* (zinc transporter ZNT7), the downregulation of which leads to cellular zinc deficiency and DNA damage; and *HIF1α,* which plays a key role in the adaptation of cells to hypoxia [4].

Transcriptional activity of AR-V7 requires dimerization. Like many other AR-Vs, AR-V7 is capable of forming heterodimers with AR-FL predominantly in the nucleus, inducing its nuclear localization in the absence of androgens. It has been shown that the FxxLF motif, which mediates N/C-terminal interaction, is crucial for the formation of such heterodimers, while the D-box within the DBD is required for AR-V7 homodimerization and its nuclear transport [75]. Furthermore, AR-V7 can heterodimerize with other receptor variants, in particular with AR-V4 and AR-V6; in addition, AR-V7 homodimerization has been demonstrated, and interaction with AR-FL is not a prerequisite for initiating the AR transcriptional program [75]. Taken together, given that in clinical settings AR-V7 is almost always co-expressed with AR-FL, it can be assumed that both heterodimers and homodimers, which possess distinct interactomes and activate different transcriptional programs, are simultaneously formed in cells.

Most AR-V7 binding sites overlap with those of AR-FL and are predominantly located in enhancers enriched for ARE and FOXA1 motifs; at the same time, 12.8% of sites are unique to AR-V7, are localized in promoters, and are enriched for the ZFX motif [76]. In addition to ZFX, the coactivator BRD4 is involved in the non-canonical regulatory function of AR-V7, providing activation of a subset of specific targets and epigenetically upregulating HOXB13 expression [77]. At the same time, AR-V7 retains the ability to interact with HOXB13, which ensures partial overlap of its transcriptional programs with AR-FL and regulates the diversity of its own cistrome in CRPC [77,78]. Notably, AR-V7 demonstrates increased binding to NCoR corepressors, which affect chromatin remodeling via the H3K27ac mark, and reduced affinity for the FOXA1 protein compared with AR-FL [79].

Despite substantial overlap between AR-V7 and AR-FL transcriptional programs, a number of studies have identified genes preferentially or specifically regulated by AR-V7. However, the composition of such signatures significantly depends on the cellular model and experimental approach, and therefore a universal AR-V7-specific transcriptional program has not yet been defined [4]. Among the most reproducible potential AR-V7 targets, the following positively regulated genes should be highlighted: *EDN2*, encoding the vasoconstrictor peptide endothelin-2 and promoting tumor cell survival, migration, and invasion; *ETS2*, regulating angiogenesis, cell cycle, and proliferation; *UBE2C*, a component of the APC/C complex that inactivates the M-phase checkpoint; *CDH2*, encoding N-cadherin and promoting EMT and metastasis; and *CDK1*, a cell-cycle regulator that directly phosphorylates AR at serines (Ser) 81 and 515. Reliably downregulated targets include *B4GALT1*, *SLC30A7*, and *HIF1α*, mentioned earlier [4]. Thus, through activation of a unique set of oncogenic target genes and simultaneous suppression of a number of anti-oncogenic factors, AR-V7 makes an independent contribution to the formation of an aggressive and therapeutically resistant PCa phenotype.

### 2.3. Dysregulation of AR Transcriptional Activity at the Intersection of Key Oncogenic Pathways

**Figure 1:**
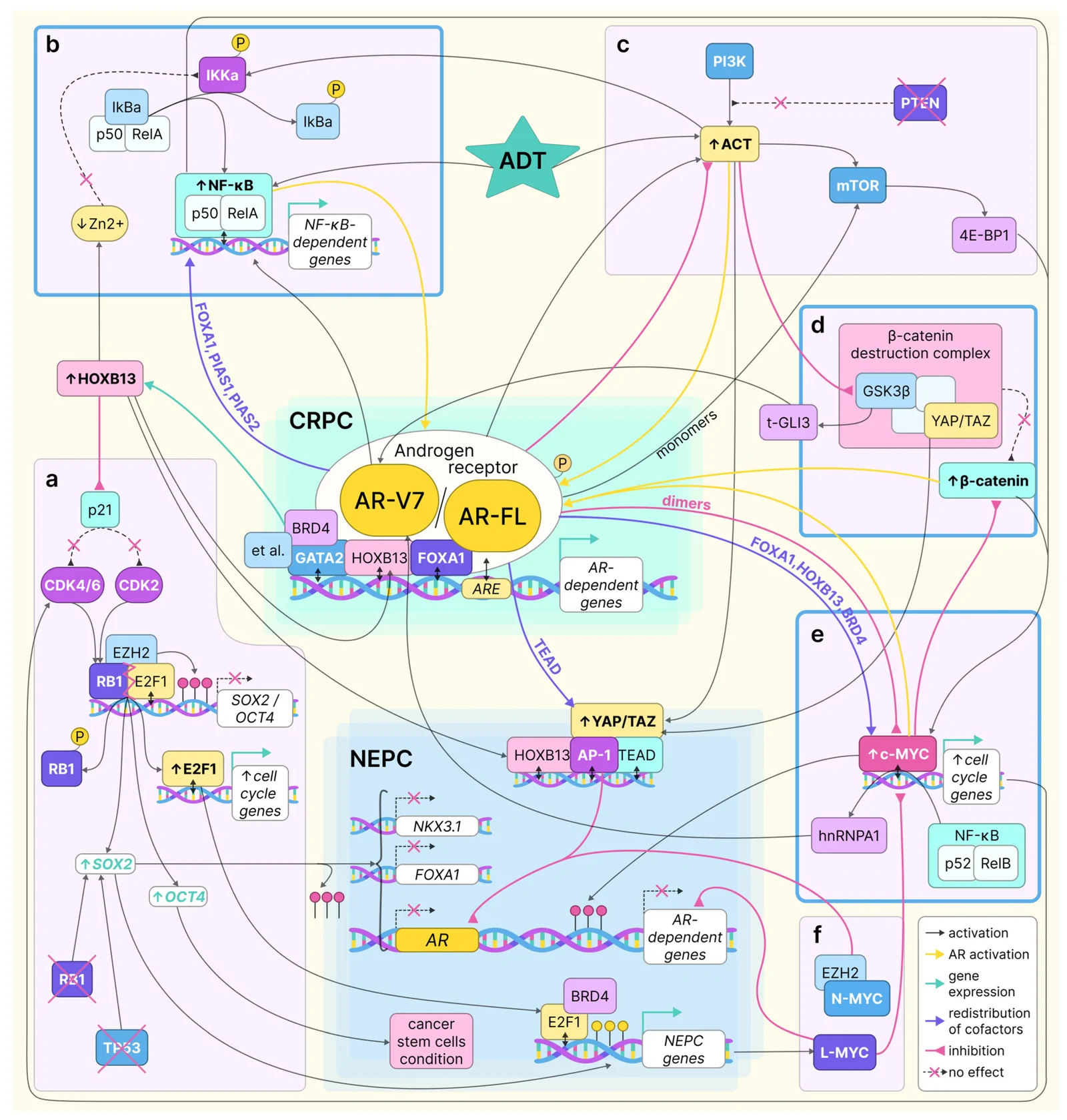
Changes in androgen receptor (AR) transcriptional activity resulting from the intersection of oncogenic pathways RB1/E2F, NF-κB, PI3K/AKT/mTOR, WNT/β-catenin, Hippo, c-MYC, and N-MYC/L-MYC in prostate cancer (PCa). (**a**) Hyperphosphorylation or deletion of RB1 derepresses E2F1, increasing expression of cell-cycle genes as well as *SOX2* and *OCT4* genes, whose expression induces stem cell properties and leads to acquisition of a neuroendocrine phenotype of prostate cancer (NEPC). HOXB13 promotes disruption of the RB1–E2F1 complex through suppression of p21 and also alters its protein interactions as the disease progresses—from interaction with AR in castration-resistant prostate cancer (CRPC) to binding with AP-1/YAP/TEAD in AR-negative NEPC, ensuring further cell proliferation and plasticity. (**b**) HOXB13 also promotes NF-κB activation, stimulating invasiveness and PCa progression. At the same time, the relationship between NF-κB and AR is complex and context-dependent: they can either activate each other or mutually suppress each other, including through redistribution of cofactors. (**c**) PTEN loss and PI3K/AKT/mTOR hyperactivation during androgen-deprivation therapy (ADT) lead to phosphorylation and androgen-independent activation of AR. (**d**) PI3K/AKT-mediated activation of the WNT/β-catenin pathway enhances AR transcriptional activity through direct interaction of β-catenin with AR. Dysregulation of the Hippo pathway resulting from hyperactivation of PI3K/AKT and WNT/β-catenin promotes nuclear accumulation of YAP/TAZ, where they compete with AR for the TEAD cofactor and suppress the AR-dependent program, contributing to an aggressive NEPC phenotype. (**e**) Overexpression of c-MYC, induced by the NF-κB, PI3K/AKT/mTOR, and WNT/β-catenin axes, promotes proliferation, invasion, and metabolic reprogramming of PCa cells. The balance between activation and repression of c-MYC by AR is determined by cofactor redistribution. At the same time, c-MYC overexpression suppresses the canonical AR transcriptional program through chromatin reprogramming at AR-dependent genes, shifting the PCa phenotype toward aggressive AR-independent proliferation. Notably, AR dimers suppress c-MYC and proliferation, whereas AR monomers (active at low androgen levels or during ADT) non-genomically activate mTOR and enhance cell-cycle gene expression, promoting proliferation and neuroendocrine transdifferentiation. (**f**) PCa progression is accompanied by a “MYC switch,” in which N-MYC mediates epigenetic suppression of AR, while L-MYC suppresses AR signaling and c-MYC expression, consolidating the neuroendocrine phenotype. Taken together, these interactions are highly complex and interconnected, forming a multifaceted signaling network rather than linear pathways. Consequently, the evolution of prostate cancer toward CRPC and NEPC is a non-linear, plastic process driven by dynamic crosstalk and compensatory feedback mechanisms among these oncogenic axes. Note: the signaling pathways presumably linking AR and CDK8/19 are outlined in the blue frame.

Progression of PCa from the androgen-dependent stage to CRPC and, especially, to NEPC is characterized by fundamental reprogramming of the transcriptional network, the central element of which is the androgen receptor. The decline in AR transcriptional activity occurs as a result of complex inter-signaling interactions. Dysregulation of key oncogenic pathways—RB1/E2F, PI3K/AKT/mTOR, WNT/β-catenin, Hippo, and various TFs (NF-κB, YAP/TAZ, MYC, and others) —triggers a cascade of epigenetic and transcriptional events that do not merely abolish AR function but actively shape a new, aggressive transcriptome (Figure 1). In this section, we will review the main molecular mechanisms that lead to inactivation of the AR transcriptional program and promote malignant transformation of PCa cells.

#### 2.3.1. Inactivation of the RB1–E2F Complex as a Trigger for AR Suppression and Cellular Plasticity

The retinoblastoma protein RB1 forms the RB1–E2F suppressor complex, which represses transcription of genes mediating DNA synthesis and cell-cycle progression. Hyperphosphorylation of RB1 by CDK4/6 or CDK2 disrupts the interaction of RB1 with E2F, activating E2F-mediated transcription of cell-cycle genes and promoting cell proliferation.

*RB1* deletion is observed in 10–15% of CRPC cases and is a molecular factor closely associated with adverse clinical outcomes in patients [80,81]. Concurrent loss of the tumor suppressor *TP53* (approximately 50% of mCRPC cases [82] leads to resistance to ADT, particularly to enzalutamide, and to increased cellular plasticity, associated in part with elevated SOX2 expression [83,84]. RB1 is known to interact with regulatory elements in the *SOX2* and *OCT4* loci, promoting their inactivation in differentiated cells, possibly through the formation of a ternary RB1/E2F1/EZH2 complex that trimethylates the H3K27 site [85,86]. The increased SOX2 expression resulting from RB1 phosphorylation or loss mediates global hypomethylation of H3K4me1/2 and H3K9me1/2, likely through upregulation of the demethylase LSD1, which leads to suppression of *FOXA1*, *NKX3.1*, *AR*, and its target genes. However, increased SOX2 expression alone is capable of stimulating expression of some neuroendocrine markers but not global acquisition of the NEPC phenotype, which apparently requires additional aberrations, such as loss of *TP53* and *RB1*, *BRN2* expression, or *REST* silencing [87,88]. In turn, OCT4 is associated with maintenance of the cancer stem cell state and enhanced metastatic potential in CRPC and NEPC [89]. Recently, it was shown that enzalutamide therapy can also promote increased SOX2 and OCT4 expression by inhibiting AR activity and, consequently, increasing *SALL4* expression, thereby inducing stem cell properties and enhancing the invasive capacity of PCa cells [90]. Notably, BRD4, interacting with E2F1, recognizes the active enhancer mark H3K27ac and activates the transcriptional program of cellular plasticity and proliferation in NEPC, which is normally suppressed by AR [91].

The function of HOXB13, which is overexpressed in CRPC, also appears to change depending on androgen levels. At low or absent androgen levels, HOXB13 suppresses p21 expression, increasing CDK2 and CDK4/6 activity, which activates the E2F signaling pathway and enhances androgen-independent tumor cell growth [92]. Furthermore, HOXB13 positively regulates the expression of other androgen-independent genes to activate cell-cycle programs, for example, the zinc transporter *ZnT4* gene. The resulting decrease in intracellular zinc concentration stimulates NF-κB-mediated signaling and increases the invasive capacity of PCa cells [93]. Interestingly, HOXB13 alters its protein interactions as PCa progresses. In normal prostate tissue, HOXB13 interacts with the tumor growth suppressor MEIS1 [94,95], whereas in early-stage PCa it begins to actively interact with AR and its constitutively active variants [77,78]. Currently, a growing body of research confirms that HOXB13 continues to be expressed in AR-negative PCa tumors, altering its cistrome and interactome through interaction with AP-1, thereby sustaining the proliferation and further neoplastic transformation of cells [31,96]. However, this statement is less applicable to neuroendocrine phenotypes, in which HOXB13 expression is nevertheless reduced [97]. In turn, AP-1, acting in concert with YAP, TAZ, and TEAD factors, drives large-scale epigenetic reprogramming of differentiated prostate cells followed by dedifferentiation into cancer stem cells, which also ensures cellular plasticity [98].

#### 2.3.2. Cross-Regulation of AR and NF-κB: From Transcriptional Interference to Context-Dependent Modulation

In turn, increased constitutive NF-κB activity in CRPC contributes to disease development and progression by regulating the expression of genes involved in preventing apoptosis, proliferation, and angiogenesis, leading to tumor invasion and metastasis [99]. According to some data, there is a positive correlation between NF-κB and AR expression in PCa cell lines and xenograft models: NF-κB can directly regulate the expression of AR-FL and AR-V7, modulating their activity [100,101,102]. It has been shown that restoration of ADT sensitivity upon NF-κB inhibition is largely associated with decreased AR-V7 expression [103]. It has also been established that AR-V7, by increasing *MALT1* expression and subsequent enhancement of nuclear translocation of NF-κB subunits p50 and p65, stimulates NF-κB activity itself [104].

However, other researchers have shown that while androgen stimulation increases the NF-κB cistrome, NF-κB stimulation with TNF-α leads to suppression of the AR cistrome. The authors found no mutual influence of NF-κB and AR on chromatin but observed redistribution of cofactors FOXA1, PIAS1, and PIAS2, which promotes exposure of latent NF-κB chromatin-binding sites and masking of AR chromatin-binding sites. As a result, a fundamentally new transcriptome is formed, including gene programs associated with cell migration and invasiveness [105]. A recent study claims that inhibition of AR signaling, including during ADT, leads to upregulation of NF-κB, activating cell survival mechanisms [106]. The counter-regulatory relationship between NF-κB and AR is corroborated by other studies. For example, forced expression of AR in the AR-negative PC3 cell line led to reduced levels of the NF-κB subunit p65, decreased its nuclear localization, reduced expression of NF-κB anti-apoptotic target genes, and consequently resulted in growth arrest and apoptosis [107]. Thus, contrary to the earliest notions of simple transcriptional interference, the relationship between AR and NF-κB is considerably more complex due to the involvement of other proteins in their cross-interactions, and its nature likely changes dynamically during PCa progression.

#### 2.3.3. PI3K/AKT/mTOR, WNT/β-Catenin, and Hippo Pathways as Drivers of AR Independence

Loss of PTEN, detected in 30–50% of CRPC tumors, leads to hyperactivation of the PI3K/AKT pathway, which critically participates in the regulation of proliferation and metabolism of cancer cells, in particular through the mTOR pathway as a key downstream effector [108]. Activation of PI3K/AKT leads to enhanced NF-κB activation as a result of AKT-mediated phosphorylation of IKKα [109]. At the same time, AR activity and the PI3K/AKT pathway are regulated by a feedback mechanism. AR regulates PI3K/AKT activity by inducing *FKBP5* expression, which stabilizes PHLPP for subsequent inhibition of AKT, and by suppressing transcription of *PLEK2*, which is required for AKT activation and stabilization [110,111,112]. However, during ADT, AR inhibition leads to hyperactivation of PI3K/AKT/mTOR, and AKT phosphorylates AR, activating it in an androgen-independent manner, which promotes therapy resistance [113,114]. In turn, PTEN loss leads to suppression of AR signaling through activation of EGR1, AP-1 (c-JUN), and EZH2, as well as degradation of NKX3.1, which is involved in the regulation of AR-dependent gene expression [110,115].

Furthermore, the PI3K/AKT pathway in PCa cells can promote activation of the WNT/β-catenin pathway through various mechanisms: for example, via AKT-mediated inactivation of GSK3β, the main component of the β-catenin destruction complex, allowing its accumulation in the cytoplasm and subsequent nuclear translocation [113]. In the nucleus, β-catenin acts as a coactivator of AR, physically interacting with its LBD and enhancing its transcriptional activity even under low androgen concentrations, thereby promoting castration resistance [116]. However, it has also been shown that in combination with PTEN gene loss, the non-canonical ligand WNT5a triggers suppression of AR expression and maintains expression of the c-MYC oncogene, which also leads to CRPC development [117].

An important regulatory node connecting PI3K/AKT/mTOR, WNT/β-catenin, and AR is the Hippo signaling pathway and its effectors—the transcriptional coactivators YAP and TAZ. Normally, YAP/TAZ activity is suppressed by the Hippo kinase cascade (MST1/2, LATS1/2), but in PCa this pathway is frequently dysregulated. PI3K/AKT activation can lead to phosphorylation and inhibition of MST1, which weakens the Hippo suppressive signal and promotes YAP nuclear accumulation [118,119]. A key mechanism linking Hippo and WNT signaling is the involvement of YAP and TAZ as components of the β-catenin destruction complex. Upon WNT signaling activation, YAP and TAZ dissociate from the destruction complex and translocate to the nucleus, where they activate transcription of their target genes in complex with TEAD factors [120]. In AR-positive PCa, YAP can antagonize AR-mediated transcription by competing with AR for binding to the common cofactor TEAD [121]. Thus, dysregulation of the Hippo–YAP/TAZ axis, along with hyperactivation of PI3K/AKT/mTOR and WNT/β-catenin, represents yet another mechanism contributing to suppression of the AR-dependent program and acquisition of a more aggressive, castration-resistant phenotype by cells.

The PI3K/AKT and WNT/β-catenin pathways also converge on non-canonical regulation of Shh signaling: Wnt/β-catenin, via GSK3β-dependent phosphorylation, participates in GLI3 processing [122], while the PI3K/AKT pathway, through the AKT1–GLI3–VMP1 axis, directs GLI3 toward autophagy [123]. The proposed GSK3β–AR–GLI3 feedback model in PCa postulates that GSK3β promotes the formation of a truncated t-GLI3 form that preferentially binds AR-V7 (more than with AR-FL), sustaining non-canonical Shh signaling activation, reinforcing the AR-dependent gene expression program, and driving castration-resistant PCa growth [124,125,126].

#### 2.3.4. c-MYC as a Central Hub of Reprogramming—Displacement of Canonical AR Activity

The frequency of amplification of the 8q24 region, where the *MYC* gene is located, reaches 80% of cases in mCRPC [127]. Overexpression of c-MYC promotes uncontrolled proliferation through direct activation of cell-cycle gene transcription (*CCND1*, *CCNE1*, *CDK4*, and others) and repression of CDK inhibitors (p21, p27) [128], enhances migratory and invasive potential via suppression of E-cadherin, and also drives profound metabolic reprogramming, including aerobic glycolysis and lactate accumulation in the microenvironment [129,130]. A mathematical model of the dynamic interrelationships between AR, c-MYC, PI3K/PTEN, and NF-κB/IκB also predicts an increase in c-MYC activity upon increased NF-κB activity following AR inhibition [106]. This may be explained by the fact that c-MYC expression can be induced by NF-κB [131], and IKKα, which activates NF-κB, participates in c-MYC stabilization, enhancing its transcriptional activity [132]. Interestingly, the alternative NF-κB2/p52 pathway stimulates the generation of the AR-V7 splice variant by regulating the expression of the splicing factor hnRNPA1 via c-MYC [133]. At the same time, activation of PI3K/AKT/mTOR also leads to increased activity and stability of c-MYC, in particular through suppression of GSK3β-mediated phosphorylation of c-MYC, which leads to its ubiquitination [134,135]. An additional intersection node is mTORC1-dependent translation of *MYC* mRNA through phosphorylation of 4E-BP1 [136]. As a result, the circuit closes: β-catenin and NF-κB ensure *MYC* transcription, and mTORC1 ensures its efficient translation, while c-MYC itself enhances transcription of *AXIN2* and *DKK1*, inhibiting the WNT pathway via negative feedback [137], indicating the existence of a delicate balance between activation and restriction of proliferative signaling depending on the disease stage.

At the same time, the dynamics of the relationship between the c-MYC oncogene and AR do not represent linear activation but rather a context-dependent equilibrium, the disruption of which determines the transition from therapy-sensitive PCa to NEPC. Thus, in normal prostate epithelial cells, ligand-activated AR acts as a growth suppressor, repressing *MYC* transcription through the formation of an inhibitory AR/β-catenin/TCF-4 complex at the gene enhancer [138]. However, as PCa malignancy increases, a fundamental switch occurs: AR acquires the ability to stimulate *MYC* expression, launching an oncogenic program [139]. It has been shown that AR, by binding to various binding sites in the 8q24-*MYC* locus, can mediate both moderate activation and repression of *MYC*. The final level of *MYC* expression depends on the redistribution of cofactors FOXA1, HOXB13, and BRD4 among these DNA sites [140]. Thus, upon loss of AR activity as a result of therapy or natural PCa progression, this balance shifts toward uncontrolled c-MYC activation, which facilitates tumor transition to an aggressive, AR-independent phenotype [141].

Some studies demonstrate that c-MYC plays a positive regulatory role in maintaining expression of both AR-FL and constitutively active splice variants (primarily AR-V7) in PCa cells [133,142]. However, it has also been shown that c-MYC overexpression plays a critical role in suppressing canonical AR transcriptional activity. For instance, it was established that c-MYC and AR share one-third of common chromatin-binding sites with enhancer properties, and c-MYC overexpression partially reprograms AR chromatin by redistributing histone marks H3K4me1 and H3K27me3, resulting in suppression of classical AR-dependent genes such as *KLK3* and *GNMT* [143]. Integration of single-cell transcriptomics data with ChIP-seq results showed that c-MYC binds to the promoters of AR-inducible genes, provoking an increase in promoter-proximal pausing of RNA Pol II, thereby blocking productive elongation of these genes, which allows it to simultaneously activate its own transcriptional program, conferring a more aggressive and proliferative phenotype upon cells [144]. For example, it is noteworthy how AR and c-MYC are involved in the regulation of polyamine synthesis in PCa. AR in the prostate activates the polyamine synthesis program by regulating the expression of key enzymes ODC1 and AMD1, while c-MYC together with the PI3K/AKT pathway optimizes this metabolic program, enhancing spermidine synthesis to activate eIF5A, which stimulates the proliferation, angiogenesis, invasion, and metastasis of PCa [145].

It is also interesting that, according to recent studies, monomeric and dimeric forms of AR are likely capable of initiating different biological programs in the cell, in particular, differentially affecting c-MYC activity [146]. It was shown that at low androgen doses, ligand-bound AR is predominantly localized in the cytoplasm as a monomer, where it non-genomically activates the mTOR signaling pathway. This also leads to enhanced RB1 phosphorylation and increased expression of cell-cycle genes *E2F1* and *FOXM1*, triggering cancer cell proliferation. When androgen levels rise, AR dimers form in the nucleus, and the expression of canonical target genes increases, ensuring maintenance of the differentiated cell phenotype. Furthermore, AR dimers suppress *MYC* expression, promote RB1 dephosphorylation, and reduce expression of G1/S transition genes, thereby suppressing cell proliferation [146]. Consistent with these data is the effect of supraphysiological androgen doses (SPA) in suppressing PCa cell proliferation: presumably, AR dimers repress *MYC* expression, leading to suppression of E2F1 signaling via the DREAM complex, consisting of RB family proteins [147]. Notably, many existing ADT approaches disrupt the favorable activity of the dimeric AR form and permit increased activity of the monomeric form, resulting in increased cell proliferation and enhanced propensity for neuroendocrine transdifferentiation. In this regard, a revision of therapy toward facilitating AR dimerization may be required [146].

#### 2.3.5. MYC Family Switch—A Paradigm Shift in Neuroendocrine Transdifferentiation

Transcription factors of the MYC family are important oncogenic factors in neuroendocrine tumors of various etiologies [148]. Progression of PCa is accompanied by a dynamic rearrangement of expression among members of the MYC family, each of which contributes specifically to the regulation of AR transcriptional activity. Whereas overexpression of the classical c-MYC, which predominates in CRPC, suppresses the AR transcriptional program without reducing AR expression levels, progression to NEPC is characterized by a qualitative shift. N-MYC, whose gene *MYCN* is amplified in approximately 40% of NEPC cases, mediates epigenetic reprogramming through interaction with EZH2, leading to suppression of AR signaling and activation of neuroendocrine programs [149]. At the same time, amplification of *MYCL* in clinical prostate cancer samples was discovered relatively recently [150], but the functional role of L-MYC in NEPC remained unstudied until recently. However, as shown in a new study, L-MYC performs a unique switch role: its expression is significantly upregulated in NEPC, whereas MYC expression is simultaneously suppressed, a phenomenon described as the “MYC family switch”. Functionally, L-MYC suppresses AR signaling, induces neuroendocrine reprogramming, and remodels cytoskeletal and adhesion pathways associated with cellular plasticity, whereas its knockdown restores c-MYC expression. Moreover, elevated L-MYC expression in NEPC is driven not so much by genomic amplification as primarily by a favorable epigenetic landscape, and it is associated with the transcriptional programs of ASCL1 and INSM1 [151]. Thus, the relationships between AR and different members of the MYC family constitute a context-dependent regulatory network in which functional substitution of c-MYC by N-MYC and L-MYC ensures sustained suppression of AR-dependent programs and consolidation of neuroendocrine identity during PCa progression.

Ultimately, PCa progression entails the formation of a complex regulatory network in which suppression of AR transcriptional activity results not from a single event but from coordinated action of multiple signaling pathways—from E2F derepression to antagonism by the MYC family. Thus, changes in AR activity are inextricably linked to redistribution of cofactors and chromatin remodeling at the level of enhancers and promoters. In this context, regulatory complexes capable of integrating multiple oncogenic signals and modulating RNA Pol II activity come to the forefront. For instance, accumulating evidence now indicates that the interaction between AR and c-MYC, as well as the implementation of their oncogenic transcriptional programs, is influenced by the activity of various transcriptional CDKs, such as CDK7, CDK8/19, and CDK9 [152]. In the following chapter, we will examine in detail the role of CDK8 and CDK19 kinases, which, acting as key modulators of this process, represent a promising target for overcoming resistance in combination therapy for PCa.

## 3. CDK8/19 as One of the Key Players in Transcriptional Reprogramming in Prostate Cancer

### 3.1. General Mechanisms of CDK8/19-Mediator Functioning

The transcriptional activity of genes depends not only on the repertoire of expressed TFs but also on chromatin state and the composition of regulatory complexes at promoters and enhancers. Thus, housekeeping genes are characterized by maintenance of an open chromatin state, owing to which their promoters remain constitutively accessible to the transcriptional apparatus. It is precisely this accessibility that allows the core Mediator (cMED) to stably interact with the preinitiation complex (PIC), RNA Pol II, and general TFs, thereby sustaining continuous transcription cycles. However, context-dependent genes are regulated differently: their expression is activated in response to hormonal, stress, differentiation, or oncogenic signals via distal enhancers and specific TFs. The activation of such genes requires chromatin remodeling, assembly of active enhancer complexes, and engagement of RNA Pol II in productive transcription.

#### 3.1.1. Structural Organization of CKM–Mediator

The CDK8/19 kinase module (CKM) comprises cyclin C (CycC) and the paralogs MED12/MED12L, MED13/MED13L, and CDK8/CDK19. These paralogs are mutually exclusive with respect to each other, but not with respect to paralogs of the other pairs, allowing for assembly of eight distinct CKM combinations, always together with CycC [153]. CKM is a tetramer in which MED12/12L allosterically activates the kinase by docking into its active center in a manner analogous to the phosphorylated T-loop, MED13/13L ensures reversible association of the module with cMED, CDK8/19 possesses kinase activity, and CycC stabilizes the catalytic domain of CDK8/19 and partially orders its structure but, unlike classical cyclins, is unable on its own (without MED12/12L) to fully activate the kinase [154].

In addition to the eight combinations of the CDK8/19 kinase module, one should also consider the variant in which the metazoan MDM2 binding protein (MTBP) acts as an allosteric activator of CDK8/19. It has a different binding surface but is mutually exclusive with MED12. Interestingly, MTBP enhances the catalytic activity of Ser5 phosphorylation in the C-terminal domain of RNA Pol II [155]. At the same time, like MED12, it shows no preference, binding to both CDK8 and CDK19 paralogs [156]. However, beyond enhanced phosphorylation, the difference between MTBP and MED12 also lies in the activation of distinct cellular programs. Thus, IFN-γ-induced phosphorylation of STAT1 Ser727 (a classical CDK8 substrate, which will be discussed further in this review) depended on MED12 but not on MTBP. By contrast, expression of the glycolysis gene *SLC2A3* depended on CDK8 and MTBP but not on MED12—meaning that the same kinase, activated by two different partners, launches two distinct transcriptional programs (N-PCa: colorectal and lung cancer) [155,157].

#### 3.1.2. Dynamics of the Transcription Cycle—From PIC to Productive Elongation

One of the key functions of the Mediator complex with CDK8/19 is the regulation of transcription cycle dynamics. In this context, CKM represents a dynamic coactivator complex that transduces regulatory signals from DNA-bound TFs to the RNA Pol II transcription machinery. In the cMED state compatible with RNA Pol II, the complex participates in PIC assembly and directly interacts with RNA Pol II and general TFs. According to the hypothesis of Allen and Taatjes, potential ncRNA–MED12 interactions may facilitate anchoring of the CDK8 module near the promoter, which could promote reversible on/off switching of CDK8 module interaction with the Mediator at the promoter, thereby regulating RNA Pol II recruitment and elongation (HYP) [158]. Cryo-electron microscopy structures of the human Mediator–PIC revealed contacts between the Head module of the Mediator and RNA Pol II and PIC components, as well as the spatial organization of the complex enabling access of TFIIH/CDK7 to the C-terminal domain (CTD) of the largest subunit RPB1 of RNA Pol II. CDK7-dependent phosphorylation of the CTD facilitates the transition of RNA Pol II from initiation to promoter escape [159,160].

A functionally distinct state is formed upon association of cMED with CKM. Structural analysis of the complete human CKM–Mediator showed that MED12 and MED13 mediate CKM association with cMED, with the extended intrinsically disordered region (IDR) of MED13 occupying cMED surfaces required for binding of MED26 and the RNA Pol II CTD (N-PCa: HEK293). Thus, the stable CKM-bound state restricts the formation of the RNA Pol II-containing PIC. At the same time, Zhao et al. demonstrated that upon CKM–Mediator binding to nuclear receptors, this state is dynamic. This binding induces rearrangement of the MED13-IDR and creates a state in which MED26 recruitment and MED26-dependent interaction of the RNA Pol II CTD with the Mediator become possible. According to the model proposed by the authors, RNA Pol II may initially interact with such an activated CKM–Mediator containing bound MED26, whereas the subsequent transition to the full Mediator–RNA Pol II holoenzyme is accompanied by CKM release (HYP). Thus, the switching of CKM–Mediator to an RNA Pol II-compatible state represents a sequential conformational rearrangement and module exchange, rather than simple static attachment of CKM to a preassembled PIC [161,162].

Subsequently, after RNA synthesis begins and RNA Pol II escapes from the PIC (promoter escape), a sequential rearrangement of the transcriptional apparatus occurs: RNA Pol II loses some interactions with general TFs and forms a complex with DSIF (SPT4/SPT5) and NELF factors. This results in the formation of the promoter-proximal paused elongation complex (PEC), comprising RNA Pol II, DSIF, and NELF [163,164]. DSIF is already a component of the early elongation apparatus; however, within the PEC together with NELF, it participates in pause stabilization. To transition to productive elongation, the PEC must undergo further rearrangement. Release of the promoter-proximal pause requires P-TEFb (positive transcription elongation factor b)—a kinase complex consisting of CDK9 and Cyclin T. MED26 represents one of the interfaces capable of linking the Mediator to the elongation apparatus: its N-terminal domain contains overlapping interaction sites with TFIID and ELL/EAF–P-TEFb-containing elongation complexes [165].

Subsequently, CDK8/19 kinase activity may contribute to pause release, i.e., the transition of RNA Pol II from pausing to productive elongation, thereby enhancing transcription of specific genes, particularly in the context of PCa. Interestingly, regulation via IFN-γ can differentially activate CDK8 and CDK19 and subsequently induce expression of distinct gene sets [166]. At the same time, the negative regulators of transcription are the MED subunits themselves, whereas CDK8 predominantly exhibits oncogenic, i.e., transcriptionally activating, effects in many cancer types [9]. Donner and colleagues showed that CDK8 is not required for initial RNA Pol II recruitment or promoter escape on serum-response genes; however, CDK8–Mediator directly interacts with P-TEFb and is required for efficient CDK9 recruitment and productive elongation [167]. This mechanism was demonstrated particularly convincingly for HIF1A-dependent transcription. Galbraith and colleagues established that prior to hypoxic activation, most HIF1A-inducible genes already contain transcriptionally active, promoter-proximally paused RNA Pol II. Under hypoxia, HIF1A recruits the CDK8-containing Mediator, including MED1 and MED26, as well as the AFF4-containing super elongation complex (SEC) and CDK9, to such loci. Knockdown of CDK8 almost completely eliminates AFF4 recruitment, substantially reduces CDK9 binding, and prevents RNA Pol II transition into the gene body, whereas TFIIH/CDK7 recruitment is preserved, indicating the absence of a primary PIC defect. Based on these data, a functional axis was proposed: HIF1A -> CDK8–Mediator -> SEC/P-TEFb -> pause release. Critically, in this system CDK8 itself is required, whereas its paralog CDK19 was not functionally equivalent to CDK8 (N-PCa: colorectal cancer) [168]. Thus, the key regulator of the transition through the promoter-proximal pause is the CDK8-containing Mediator.

The direct catalytic release of the pause is mediated primarily by CDK9: P-TEFb phosphorylates RNA Pol II and SPT5/DSIF and participates in PEC rearrangement. Subsequent rearrangement is accompanied by removal of NELF and incorporation of positive elongation factors, including PAF1C and SPT6; the structurally activated elongation complex is represented by RNA Pol II–DSIF–PAF–SPT6. Genome-wide Global Run-On and Precision Run-On sequencing (GRO-seq and PRO-seq) studies of the IFN-γ response further demonstrated that CDK8 kinase activity facilitates RNA Pol II pause release, whereas CDK19 in this system performs a distinct, predominantly kinase-independent function (N-PCa: mouse embryonic fibroblasts (MEFs), colon cancer) [166,169].

At the same time, the precise spatiotemporal organization of CKM relative to the already-formed PEC remains not fully established. Available ChIP, biochemical, and proteomic data demonstrate functional linkage of the CDK8–Mediator with SEC/P-TEFb and promoter-proximal RNA Pol II; however, a structure of the unified CKM–Mediator–SEC–paused RNA Pol II complex has not yet been obtained. Therefore, it cannot be unequivocally stated whether CKM remains associated with the same cMED during pause release, undergoes further rearrangement, or acts as a partially or fully dissociated kinase module. For HIF1A-dependent genes, the most substantiated working model involves additional HIF1A-dependent recruitment or rearrangement of the CDK8-containing Mediator at loci already containing the PEC, followed by recruitment of SEC/P-TEFb and CDK9-dependent transition of RNA Pol II to productive elongation (HYP).

#### 3.1.3. Catalytic Functions of CDK8/19—Substrate Phosphorylation

The function of CKM is not limited to structural regulation of the Mediator. Upon gene activation by signal-dependent TFs, CDK8/19 is selectively recruited to activated loci, where it phosphorylates specific TFs (STATs, SMADs, E2F1, and others), thereby enhancing transcriptional induction of silent genes [9]. In addition, CDK8/19 phosphorylates proteins involved in chromatin remodeling: in particular, analysis of CKM interaction with SWI/SNF identified three subunits—ARID1A, SMARCC2, and PBRM1—that bound to MED12 and were phosphorylated in the presence of Mediator kinases (N-PCa: intestinal epithelium) [170]. Accordingly, CDK8/19 inhibition has a substantially greater impact on transcriptional responses following serum, NF-κB, or PKC-dependent signaling than on gene expression under basal conditions, with induction of the most strongly activated genes being suppressed most markedly [9]. Thus, CDK8/19 are particularly important for enhancing and remodeling signal-inducible transcriptional programs, rather than for uniformly maintaining global transcription. In this context, CDK8/19 function as a “transcriptional reprogramming switch,” rather than as a universal activator or repressor (N-PCa: ovarian, pancreatic, and mammary cancer) [171,172].

##### Coordinated Function with NF-κB and RNA Pol II Phosphorylation

The CTD of the large subunit of RNA Pol II is a direct target of CDK8/19: phosphorylation of serine residues within the heptapeptide repeats of the CTD triggers the transition from initiation to productive transcription elongation. However, CDK8/19 possess kinase activity toward the RNA Pol II CTD primarily in vitro, whereas in cells their main contribution lies in organizing the recruitment of TFIIH/CDK7 and P-TEFb/CDK9 to genes, where these kinases perform phosphorylation of Ser5 and Ser2 of the CTD, respectively, thereby initiating the transition from initiation to productive elongation (N-PCa: colorectal cancer) [167]. Crucially, CDK8/19 carry out this phosphorylation in a gene-specific manner, preferentially affecting genes regulated by specific TFs.

CDK8/19 have been shown to function in coordination with NF-κB: inhibition of CDK8/19 suppressed RNA Pol II CTD phosphorylation at promoters of NF-κB-dependent genes, blocking productive transcription elongation. As a result of RNA Pol II phosphorylation involving CDK8/19 in response to NF-κB activation, transcriptional induction of key proinflammatory chemokines—CXCL1, CXCL2, and IL-8 (CXCL8)—occurs [171]. These chemokines possess potent protumorigenic activity: CXCL1 and CXCL2 stimulate angiogenesis, invasion, and formation of an immunosuppressive microenvironment, whereas IL-8 is a powerful proangiogenic and proinvasive cytokine [173]. Furthermore, some studies report joint recruitment of CDK8/19 and NF-κB to chromatin, viewing them as mechanistic partners. Importantly, CDK8/19 do not act as classical NF-κB activators (they do not affect its nuclear translocation or DNA-binding), but rather as coregulators of elongation: upon NF-κB activation, CDK8/19 are corecruited with it to promoters of NF-κB-dependent genes [174].

##### Phosphorylation of STAT1, STAT3, and STAT5—Regulation of Immune Response and Oncogenic Transcription

One of the most extensively studied substrates of CDK8 are proteins of the STAT family. CDK8 has been shown to phosphorylate STAT1 (Ser727), as well as the analogous regulatory serines of STAT3 (Ser727) and STAT5 (Ser726), directly following binding of these TFs to target gene promoters [175]. The effect of this phosphorylation is context-dependent and ambiguous: for STAT1, CDK8-dependent phosphorylation regulates over 40% of IFN-γ-inducible genes, including antiviral and immune programs (N-PCa: MEFs, hepatocarcinoma) [175]. Notably, there is a contradiction in the data regarding the role of CDK8 in STAT1-dependent NK-cell cytotoxicity: CDK8-mediated phosphorylation of STAT1 limits the anti-tumor activity of NK cells (N-PCa: melanoma, leukemia, and breast cancer) [176]. In the case of STAT3, CDK8 limits the residence time of STAT3 at gene promoters, fine-tuning transcriptional responses to IL-6, including regulation of Th17 differentiation [177]. The pattern of target genes regulated via STAT3 with CDK8/19 involvement predominantly includes genes sensitive to the duration and amplitude of STAT3 signaling (e.g., genes involved in cell proliferation and survival), whereas a number of classical IL-6 targets (e.g., *SOCS3*, *BCL3*) are induced by HyIL-6 but remain independent of CDK8/19 (N-PCa: CD4+ T cells) [175,177]. For STAT1, CDK8 acts as a direct kinase required for full activation of IFN-γ-dependent genes, whereas for STAT3, CDK8/19 function rather as negative regulators of promoter occupancy, limiting the duration of STAT3-dependent transcription. Beyond STAT1 and STAT3, CDK8 also phosphorylates STAT5 at Ser726, as demonstrated in cellular models of acute myeloid leukemia and other tumors; inhibition of CDK8/19 reduces STAT5 Ser726 phosphorylation and suppresses STAT5-dependent transcription; however, detailed characterization of STAT5 targets regulated by CDK8/19 remains less complete compared with STAT1 and STAT3 (N-PCa: acute myeloid leukemia; various cancers) [178,179].

##### Activation of Smad1 and Smad2/3 via Linker Region Phosphorylation

CDK8 and CDK9 have been identified as nuclear kinases responsible for phosphorylation of the linker region of Smad proteins following their activation by TGF-β and BMP receptors [180]. When receptor-activated Smad proteins (rSmads) form transcriptional complexes in the nucleus, CDK8/19 phosphorylate linker serine/threonine-rich sites (PXSP motifs), which serves as a dual “activation/destruction switch”: on the one hand, the phosphorylated linker recruits transcriptional coactivators (YAP1 for STAT1 and PIN1 for STAT3), enhancing expression of Smad-dependent genes; on the other hand, it creates sites for Smurf1/Nedd4L ubiquitin ligases, targeting Smad proteins for ubiquitin-dependent degradation (N-PCa: HEK293T) [181].

For Smad1 (BMP pathway), CDK8 phosphorylates the linker, first ensuring maximal transcriptional activity of BMP targets and then their timely shutdown through degradation of activated Smad1. The overall mechanism can be outlined as follows: first, CDK8/9 phosphorylate Ser206/Ser214, creating binding sites for YAP1 (via YAP WW domains); subsequently, CDK8/9 primes a second adjacent site for GSK3β, whose phosphorylation strengthens the binding site recognized by E3 ligases (Smurf1 in the case of Smad1) (N-PCa: various cells) [182]. From the above discussion, we know that YAP is capable of antagonizing AR-mediated transcription by competing with AR for binding to the common cofactor TEAD [121]. This indicates that CDK8-dependent phosphorylation of Smad1 not only regulates the duration of BMP signaling but also indirectly modulates AR pathway activity: by recruiting YAP1 to the phosphorylated linker, CDK8 presumably redirects a portion of the YAP1 pool toward its canonical function in Smad1 degradation, thereby reducing its availability for competition with AR over TEAD. Thus, CKM activity may act as a kind of “switch” between BMP- and AR-mediated transcriptional programs (HYP).

For Smad2/Smad3 (TGF-β pathway), CDK8-dependent linker phosphorylation at S208 and T179 (Smad3) enhances TGF-β-inducible gene expression, including genes associated with EMT. Notably, CDK8/9 primarily target Thr179, whereas Ser208 and Ser213 are secondary sites [182,183]. Significantly, CDK8/19 in conjunction with YAP1 enhances BMP4-induced EMT in tumor cells—a process critical for metastatic progression (N-PCa: ovarian, pancreatic, and mammary cancer) [172]. Specifically for Smad3, CDK8/9-dependent linker phosphorylation at Thr179 (as well as Ser208 and Ser213) enhances TGF-β-inducible gene expression but simultaneously primes the linker for subsequent phosphorylation by GSK3β and recognition by the ubiquitin ligase Nedd4L, leading to polyubiquitination and proteasome-mediated degradation of Smad3 (N-PCa: various cancers) [180,184,185].

##### Inactivation of the RB1-E2F Complex and Activation of β-Catenin

CDK8 also contributes additionally to the disruption of the functional RB1-E2F1 complex by directly phosphorylating E2F1 at S375, which reduces the ability of E2F1 to suppress β-catenin-dependent transcription and leads to cell-cycle dysregulation (N-PCa: colorectal cancer) [186]. Thus, CDK8/19-dependent dysregulation of the RB-E2F1 axis becomes an additional mechanism for sustaining proliferation amid progressive RB loss following ADT.

Interestingly, CDK8 was first identified as an oncogene when it was found to be an essential coactivator of β-catenin transcriptional activity in colorectal cancer cells (N-PCa: colorectal cancer) [186]. CDK8 enhances the nuclear transcriptional activity of β-catenin in conjunction with TCF/LEF factors, thereby inducing expression of Wnt targets, including *MYC* (N-PCa: colorectal cancer) [187]. Moreover, recent studies have shown that suppression of CDK8/19 and MED12 in PCa significantly reduces c-MYC protein expression [10,11]. Furthermore, as discussed in previous sections, β-catenin in PCa acts as a coactivator of AR [116], and c-MYC and AR are engaged in a complex regulatory relationship [142,144], and others. Thus, there are grounds to hypothesize that the CDK8–β-catenin–c-MYC–AR axis may function as a mutually reinforcing loop; however, this remains a hypothetical reconstruction: individual components have been demonstrated in different model systems, but the complete circuit has not been directly investigated in PCa cells (HYP).

##### Phosphorylation of SWI/SNF Components—Chromatin Remodeling as a Substrate Class

The primary function of the Mediator complex is to bind RNA Pol II and recruit PIC factors, and subsequently, upon association with the CDK8 module, to regulate RNA Pol II elongation and its release from pausing, including through association with SEC, thereby enabling rapid transcriptional responses. At the same time, one cannot overlook another function of the Mediator complex—its interaction with chromatin-remodeling complexes, including SWI/SNF, as a result of which the Mediator complex facilitates their recruitment and/or functioning at activated promoters and enhancers [158].

The SWI/SNF complex exists in cells in several assembly variants, primarily cBAF and PBAF, which maintain an open chromatin state. The authors [188] note that many observations raise the question of how the noncatalytic core portion of the BAF complex (for example, ARID1A, which is frequently mutated in cancer) may contribute to its molecular function at enhancers and promoters. A 2022 study directly investigated the interaction of SWI/SNF, BAF, and the Mediator complex in intestinal biology: it was found that the Mediator complex is required for ARID1A binding at enhancer regions, and that CDK8/19 not only colocalizes with SWI/SNF but also appears to act as a “guide” for positioning the complex. CDK8/19 phosphorylate ARID1A at Ser365 [170] (as well as SMARCC2 and PBRM1), as established by superimposition of the phosphoproteome with the MED12 interactome. This mechanism allows ARID1A to concentrate at approximately 1350 CDK8/19-dependent enhancers, for whose activity the kinase activity of the Mediator is required (N-PCa: intestinal epithelium). The presence of physical MED12–ARID1A interaction within the Mediator complex is a necessary condition for proper genomic targeting of the SWI/SNF complex to enhancers. Thus, loss of CDK8/19 may entail mislocalization and altered distribution of ARID1A between enhancers and promoters. It is also noteworthy that under normal conditions, CDK8/19 control *ATOH1* expression by regulating individual ARID1A-defined *ATOH1* super-enhancers 3/4 (N-PCa: intestinal epithelium) [170]. It is also interesting that ARID1A acts as an antagonist of c-MYC and PARP1 signaling pathways, coordinating proliferation control and integrity in small-cell lung cancer (N-PCa: small-cell lung cancer) [189]. This demonstrates that ARID1A performs at least two independent functions relevant to tumors—effects on enhancers and direct transcriptional suppression of oncogenes. Whether one, both, or neither of these functions are engaged in PCa remains to be determined, but this provides strong support for the importance of the noncatalytic core portion of the BAF complex (HYP).

#### 3.1.4. Phosphorylation-Independent Functions of CDK8/19

The effects of CDK8/19 on transcription discussed above are explained by the kinase activity of these proteins, namely phosphorylation of substrates such as Smad1 or components of the PIC. However, accumulating evidence indicates that CDK8/19 also possess kinase-independent functions, which may potentially influence AR signaling and PCa progression through alternative pathways, including stabilization of protein partners, regulation of transcriptional programs, or modulation of the tumor immune microenvironment (HYP).

It is known that both kinases stabilize CycC, protecting it from proteolytic degradation in a manner completely independent of catalysis: genetic knockouts and catalytically inactive mutants protect CycC as effectively as wild-type proteins (N-PCa: MEFs, colorectal and cervical cancer) [9].

Particular attention should be paid to CDK19 knockdown in SJSA osteosarcoma cells, which resulted in reduced proliferation and increased expression of p53 pathway genes; however, re-expression of both wild-type and catalytically “dead” CDK19 mutants equally restored the ability of cells to resume proliferation following p53 activation by nutlin-3, demonstrating the kinase-independent nature of this function (N-PCa: osteosarcoma) [190]. In BCR-ABL1-positive B-cell acute lymphoblastic leukemia, genetic loss of CDK8 significantly increased disease latency and blocked its maintenance, whereas pharmacological suppression of kinase activity produced only a minimal effect. Gene set enrichment analysis linked CDK8 loss to dysregulation of mTOR signaling, leading to the development of the compound YKL-06-101, which combines mTOR inhibition with CDK8 protein degradation (N-PCa: BCR-ABL1+ B-cell acute lymphoblastic leukemia) [191]. In triple-negative breast cancer cells, CDK8 knockdown deregulated 887 genes compared with only 25 upon Senexin B inhibition, with almost no overlap. The authors demonstrated that CDK8-dependent but kinase-independent regulation of EMT and expression of PD-L1 and the NKG2D ligand RAE1 forms a CDK8–PD-L1 axis that enables tumor evasion from NK-cell immune surveillance (N-PCa: triple-negative breast cancer) [192].

It should be noted that for cases where noncatalytic function has been inferred from comparison of genetic protein loss and pharmacological inhibition of catalysis (N-PCa: BCR-ABL1+ B-cell acute lymphoblastic leukemia; triple-negative breast cancer) [191,192], alternative explanations such as incomplete pharmacological suppression of kinase activity or the existence of unidentified substrates accessible only to a subpopulation of the CDK8/19 pool cannot be completely excluded. However, data obtained using catalytically dead mutants (N-PCa: osteosarcoma) [9,190], support a genuinely kinase-independent mechanism, independent of such caveats.

Taken together, these disparate yet notable findings form a new paradigm: CDK8 and CDK19 should be regarded less as kinases and more as multifunctional proteins whose role in carcinogenesis is only partially mediated by phosphorylation. However, the molecular mechanisms mediating these effects and their protein partners remain insufficiently characterized. Further work employing proteomic approaches and conditional knockout models is necessary to differentiate the catalytic and structural roles of these kinases in normal and pathological states, including in PCa.

### 3.2. Regulatory Interplay Between CDK8/19 and AR in Prostate Cancer

#### 3.2.1. Direct Link Between CKM Subunits, the Mediator Complex, and AR

The Mediator complex, and, particularly, some of its subunits, is likely involved in regulating AR activity in PCa, so they should be considered not only individually but also in terms of their interaction with coactivators.

It is known that CDK19 expression progressively increases throughout the entire oncogenic trajectory of PCa, whereas the dynamics of CDK8 are non-monotonic. Androgens differentially regulate the expression of the paralogs: AR signaling represses CDK8 and activates CDK19, resulting in decreased CDK8 and increased CDK19 expression as PCa progresses. However, both proteins are overexpressed in mCRPC [193]. In a large immunohistochemical study, nuclear expression of CDK8/19 showed the highest intensity specifically in metastatic and castration-resistant tumors. High CDK19 levels in primary tumors, independently of Gleason score and PSA, predicted disease recurrence: 5-year recurrence-free survival was 73.7%, 56.9%, and 30.4% for groups with absent, moderate, and high CDK19 expression, respectively [8]. An important aspect was the correlation of CDK19 with the proliferation marker Ki67 and nuclear AR in primary tumors [8], indirectly pointing to a functional link between CDK19-dependent transcription, AR activity, and the proliferative potential of tumor cells. Moreover, in primary tumors, CDK19 was significantly associated with ERG rearrangement and ERG expression. At the same time, CDK8 levels did not differ substantially between benign tissue and primary tumors, while a marked increase in CDK8 was observed mainly in metastases and CRPC [194]. It is also noteworthy that in another study, in clinical samples of NEPC and CRPC, the frequency of genomic amplification of *CDK8*, *CDK19*, and *CCNC* was determined to be the highest among the various cancer subtypes studied: in CRPC, the amplification frequency of one or more of these genes was 18.6% (13 of 70 samples), and in NEPC, it was 19.6% (11 of 56 samples) [195].

Thus, among unresolved questions, there remains the issue of the functional interchangeability of CDK8 and CDK19 specifically in PCa: although overall both paralogs perform qualitatively similar functions [9], clinical data suggest that CDK19 appears to be the dominant paralog in PCa, whereas CDK8 is more often considered an oncogene in colorectal cancer. This difference in paralog expression indicates that in primary PCa, the earliest dependence on the kinase module may be primarily linked to CDK19, while upon progression the contribution of both paralogs increases. This pattern is one of the starting points for direct investigation of the AR–CDK8/19 interconnection.

Notably, MED12 is also overexpressed in advanced CRPC compared to early-stage disease, and knockdown of MED12 reduces cell proliferation and S-phase entry [11]. Moreover, loss of MED12 suppressed AR directly at the protein level, not only at the level of downstream AR target genes; however, the specific mechanism of this reduction—decreased synthesis due to an AR autoregulatory loop or enhanced degradation—was not established in that study [196]. For its paralog MED12L (whose sequence is more divergent), which is also frequently amplified and overexpressed in aggressive forms of PCa, including mCRPC and BRCA2-mutant tumors [197], analogous data on AR regulation at the protein level are currently lacking.

Regarding MTBP—another CKM subunit that binds to CDK8/19 instead of MED12—it is known to associate with c-MYC through direct interaction with Tip48 and Tip49 at MYC-dependent gene promoters, thereby activating its oncogenic programs (N-PCa: various cancers) [198]. This link was also demonstrated in in vivo lymphoma studies: MTBP deficiency inhibited c-MYC-induced proliferation (N-PCa: B-cell lymphoma) [199]. The CDK8/19–c-MYC connection via MTBP could potentially explain the effect of CDK8/19 inhibition on MYC-dependent pathway activity [11], but this axis requires further investigation (HYP). In addition, some studies also mention that MTBP can function independently of its classical partner MDM2. At the same time, the MDM2 protein, stabilized by MTBP, can directly regulate AR: MDM2 acts as an E3 ubiquitin ligase for AR-FL, AR-V7, and TM4SF3 in PCa cells, and MDM2 inhibition elevates their expression levels. Separately, it should be noted that in PCa stem cells, MDM2 promotes constitutive ubiquitination and degradation of AR, maintaining low AR levels in this population [200]. Given the intricate interplay with AR E3-ubiquitination programs and the connections with CDK8/19 and c-MYC, these data allow the hypothesis that MTBP may be yet another node linking c-MYC-driven transcriptional programs with RNA Pol II output controlled by the Mediator kinase (N-PCa: various cancers, review) [198]. However, this circuit has not been directly tested in any study, and data on MTBP expression in PCa are currently absent. Moreover, since MED12 is also overexpressed in CRPC and competes with MTBP for the same pool of CDK8/19, it remains unclear whether the fraction of MTBP-associated CDK8/19 actually increases at the stage required for this mechanism (HYP).

The role of other Mediator complex subunits after chromatin remodeling and AR recruitment to distal enhancers or super-enhancers is also of interest in PCa [201]. For example, TFIIH/CDK7 is recruited and phosphorylates MED1 at threonine 1457, thereby promoting Mediator binding to AR [202]. MED1 binds to a specific N-terminal region of AR, termed the transactivation unit-1 (Tau-1), via two non-canonical α-helical motifs [203]. Following attachment, the Mediator connects the enhancer-bound AR complex to the promoter-proximal PIC, physically forming chromatin loops to bring the enhancer closer to RNA Pol II, TFIID, TFIIB, and TFIIH [201].

#### 3.2.2. Involvement of CDK8/19 in Chromatin Remodeling and Regulation of AR-Dependent Transcription in PCa

As mentioned earlier, CDK8/19 phosphorylates SMARCC2, which is part of the common structural core of the SWI/SNF (BAF) chromatin-remodeling complex, as well as ARID1A and PBRM1, which are subunit identity components of BAF variant complexes (cBAF and PBAF) that direct them to specific genomic regions. Other BAF subunits—SMARCA2/SMARCA4—are two mutually exclusive catalytic ATPases that in PCa most often show dysregulation in the form of altered expression. A striking finding is that AR^+^/FOXA1^+^ PCa cells are sensitive to degradation of SMARCA2 and SMARCA4, which rapidly compacts enhancers bound by AR, FOXA1, ERG, and MYC, displacing these factors from chromatin and suppressing their oncogenic programs, including super-enhancers that drive overexpression of *AR/FOXA1/MYC* [204,205]. Of course, at present the evidence base for an interaction between SMARCA2/SMARCA4 and CDK8/19 in PCa is insufficient, but it can be hypothesized that CDK8/19, through regulating the positioning of the complex at enhancers of AR-, FOXA1-, ERG-, and c-MYC-dependent genes via phosphorylation of the scaffold part of the module, may direct transcription of these genes (HYP). Upon CDK8/19 inhibition, recruitment or retention of SWI/SNF at the corresponding regulatory sites is likely disrupted, which may attenuate transcription of these programs. Thus, the influence of CDK8/19 on transcriptional programs may occur not through a direct kinase–substrate interaction, but via separate, parallel mechanisms of Mediator transcriptional activity and co-regulation of AR-dependent transcription through cBAF/PBAF.

In addition, it is known that ERG—an oncogenic ETS-family factor, discussed earlier in the context of AR-cistrome reprogramming—physically interacts with the SWI/SNF/BAF complex and causes its global redistribution to ETS-dependent regulatory sites, followed by ATP-dependent nucleosome remodeling by BAF [206]. Thus, ERG directly interacts with AR and simultaneously alters the chromatin landscape through BAF. AR, in turn, can bind to the MED1 subunit and recruit the Mediator to AR-regulated sites [203]. In ERG-negative ETS subtypes, another mechanism may engage the oncogenic TF network with the Mediator. High-affinity interaction with MED25 is characteristic of the ETV1/ETV4/ETV5 subfamily, with this mechanism being most thoroughly studied for ETV4 [207]. In AR-negative PC-3 cells overexpressing ETV4, about 75% of ETV4-bound sites overlapped with MED25 and were predominantly located at distal enhancers; knockdown of ETV4 or MED25 reduced expression of common target genes [207]. Consequently, different molecular variants of PCa may employ distinct mechanisms of Mediator engagement: in the AR-dependent network, the AR–MED1 contact is well established, whereas ETV1/4/5 can recruit the Mediator via MED25. This creates a possibility for convergence of various oncogenic TF networks onto a single central transcriptional node.

Against this background, CDK8/19 may represent a common regulatory layer for distinct oncogenic transcription factor networks. Direct evidence for a functional relationship between CKM and SWI/SNF has been obtained outside the PCa context, where CDK8/19 regulate the genomic positioning of the SWI/SNF subunit ARID1A (N-PCa: intestinal epithelium) [170]. In PCa, ERG interacts with BAF chromatin-remodeling complexes, whereas AR can recruit the Mediator through MED1 [203,204,205,206]. Our recent integrative in silico analysis of the *AR* promoter revealed overlapping AR and ERG ChIP-seq signals in the proximal promoter of VCaP cells, alongside distal FOXA1-associated regions and context-dependent H3K27ac enrichment [208]. These observations identify candidate regulatory regions for experimental analysis of the proposed AR/ERG–chromatin-remodeling network. The proposed ETS/AR–SWI/SNF–CKM–Mediator axis remains a mechanistic hypothesis in PCa (HYP). Its validation in ERG-positive models such as VCaP requires separate CDK8 and CDK19 perturbations combined with analyses of chromatin occupancy and transcriptional activity.

Furthermore, CDK8/19 appears to rarely act as the sole driver of a particular transcription step, but rather systemically modulates the activity and localization of other kinases (CDK7, CDK9, and to some extent CDK12/13), thereby determining which genes will be efficiently transcribed and which will be repressed. We have already described how CDK8 can regulate CDK7, so we will next examine its effect on CDK9 and CDK1/2/4/6. Direct data showing that CDK8/19 regulates CDK1/2/4/6 kinases have not been reported in the literature; however, as described earlier, CDK8/19 modulates CDK7 at the transcriptional level, and CDK7 in turn can phosphorylate the T-loop of CDK1, CDK2, and CDK4/6, thereby activating them in the cell cycle. Classically, CDK8/19 is thought to phosphorylate and affect E2F1, but it may indirectly influence the second component of the complex—RB—since CDK7 phosphorylates RB and releases E2F1 [209]. In this case, CDK8/19 should be considered not as a direct regulator of kinase activity but as a transcriptional regulator. At the same time, in the case of CDK9, the role of CDK8 is rather specific. As shown in the study in Reference [167], CDK8–Mediator regulates precise steps in RNA Pol II elongation complex assembly, including recruitment of P-TEFb and BRD4. Moreover, upon CDK8 knockdown, recruitment of CDK7 and CDK9 to the FOS and EGR1 loci is disrupted (shown outside the PCa context). At the same time, as mentioned earlier, it is separately known that BRD4 is required for AR-V7-dependent transcription and CRPC growth—a direct link between these two observations in PCa cells has not been tested, but it represents an interesting hypothesis (HYP) [210].

Thus, the body of evidence reviewed allows for the proposition that CDK8/19 may act as an integrator of AR-dependent transcriptional programs, linking chromatin remodeling through SWI/SNF with recruitment of P-TEFb/BRD4 and RNA Pol II pause release (HYP). This is particularly important in AR-V7-positive CRPC, where BRD4 is required for maintaining oncogenic transcriptional networks that bypass classical androgen regulation [77]. On this basis, inhibition of CDK8/19 could simultaneously attenuate both AR-FL- and AR-V7-dependent programs, making this module a promising target for overcoming resistance. Direct verification of this integrative model in PCa cells, however, is still lacking.

**Figure 2:**
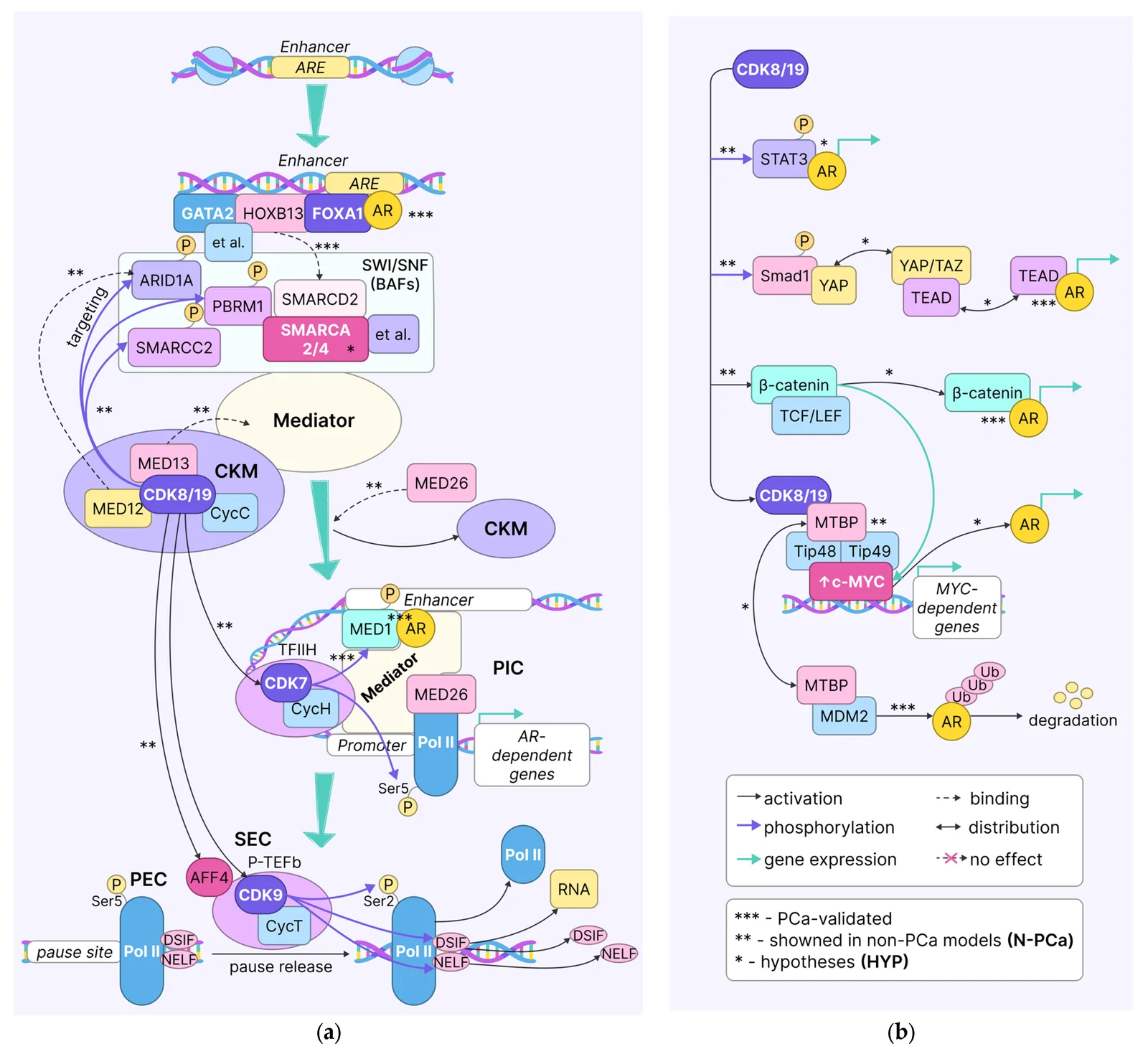
Proposed mechanisms of the influence of cyclin-dependent kinases 8 and 19 (CDK8/19) on androgen receptor (AR) transcriptional activity in prostate cancer (PCa). (**a**) Based on fragmented data regarding the established functions of CDK8/19 in cells, a mechanism is proposed for the involvement of the CDK8/19 kinase module (CKM) in chromatin remodeling and regulation of AR-dependent transcription in PCa. It is known that the SWI/SNF chromatin-remodeling complex can be recruited to enhancers of AR-dependent genes through interaction of HOXB13 and SMARCD2. Interaction of SMARCA2/4 subunits with enhancers of AR, FOXA1, ERG, and c-MYC-dependent genes in AR^+^/FOXA1^+^ PCa cells is also presumptive. In addition, it has been established that CDK8/19, as part of the Mediator complex, can phosphorylate certain SWI/SNF subunits, including ARID1A, which is required for proper genomic targeting of the complex to enhancers. Subsequent formation of the preinitiation complex (PIC) is accompanied by release of CKM, MED26-dependent interaction of the RNA polymerase II (RNA Pol II) C-terminal domain (CTD) with the Mediator, and formation of a chromatin loop to bring the enhancer closer to the promoter. At the same time, CDK7 within TFIIH phosphorylates MED1, enhancing Mediator binding to AR, and also phosphorylates the RNA Pol II CTD at Ser5, facilitating the transition from transcription initiation to PIC escape. In turn, CDK9 within P-TEFb phosphorylates the RNA Pol II CTD at Ser2, inducing pause release. Meanwhile, CDK8/19 modulates the activity and localization of CDK7 and CDK9 at all stages of transcription, determining which genes will be efficiently transcribed and which will be repressed. (**b**) CDK8/19 can influence AR transcriptional activity through phosphorylation of several substrates. CDK8/19 can phosphorylate STAT3, fine-tuning transcriptional responses to IL-6, whereas the STAT3 ligand independently activates AR. CDK8-mediated phosphorylation of the linker region of Smad1 promotes its binding to YAP, presumably reducing competition between YAP and AR for the common cofactor TEAD, which may also enhance AR transcriptional activity. In addition, CDK8 also potentiates the transcriptional activity of β-catenin, which is a coactivator of AR and can induce expression of c-MYC, which exists in a complex regulatory relationship with AR. Furthermore, the possible competition between CDK8/19 and MDM2 for MTBP is noteworthy, whereby redistribution of the MTBP pool may lead to activation of c-MYC-dependent programs or to AR degradation. Importantly, all mechanisms illustrated in this figure for the influence of CDK8/19 on AR transcriptional activity represent a reconstruction and require further investigation in PCa cells. The asterisks indicate the level of evidence for the depicted mechanisms, which are classified as follows: (***) PCa-validated mechanisms established in PCa models; (**) mechanisms demonstrated in non-PCa models (N-PCa); and (*) mechanistic hypotheses (HYP) representing logical inferences that require further experimental validation in PCa cells.

The proposed pathways of AR transcriptional activity regulation via CDK8/19 activity, based on the information presented in Section 2.1 and Section 2.2, are summarized in Figure 2.

#### 3.2.3. Impact of CDK8/19 Inhibition on AR Transcriptional Activity

The mechanisms described above may prospectively explain the relatively recent proposal by Offermann et al. that CDK8/19 partially mediates its pro-oncogenic effects through modulation of AR and that combination therapy, pairing AR blockade with CDK8/19 inhibition, could represent a strategy to enhance treatment efficacy.

In the study by Offermann et al., the combination of bicalutamide and a CDK8/19 inhibitor significantly reduced the viability of androgen-independent LNCaP-Abl cells after prolonged treatment [211]. Later key studies have also demonstrated that inhibition of components of the Mediator complex kinase module represents a promising strategy to overcome therapy resistance in PCa. In the work of Li et al., genetic and pharmacological inactivation of CDK8 and CDK19 was performed in in vivo CRPC xenograft models. The authors showed that CDK8/19 inhibition reverses the CRPC phenotype and, in combination with castration, induces tumor regression. At the same time, *MYC* gene expression decreased upon castration and castration combined with CDK8/19 inhibition, but not upon CDK8/19 inhibition alone, indicating that CDK8/19 presumably exerts its effects downstream of the c-MYC signaling pathway [10]. In the work by Andolfi et al., performed predominantly on LNCaP and 22Rv1 cell lines, both MED12 (knockdown) and CDK8/19 kinases (pharmacological inhibition) were targeted. The authors found that MED12 suppression significantly reduces c-MYC protein expression and its signaling pathway activity and decreases PSA expression and other AR target genes, as well as AR-V7 in the enzalutamide-resistant 22Rv1 cell line. Critically, both MED12 knockdown and CDK8/19 inhibition in combination with enzalutamide produced an additive effect, reducing AR activity and cell proliferation [11]. These same effects upon MED12 knockdown were subsequently confirmed in enzalutamide-resistant LNCaP-Abl EnzR and DuCaP EnzR cell lines [196].

Thus, both approaches—pharmacological inhibition of CDK8/19 catalytic activity and knockdown of the structural component MED12—lead to similar phenotypic outcomes (suppression of c-MYC and AR pathways); however, given the possible kinase-independent effects of CDK8/19 demonstrated in the previous section, it cannot be excluded that these two interventions act through partially distinct molecular mechanisms. At the same time, a certain contradiction is observed regarding MED12: a number of studies have shown that genetic alterations in MED12 or its depletion, on the contrary, promote the development of CRPC through hyperactivation of the Shh-dependent GLI3 signaling pathway, which, as mentioned earlier, selectively binds AR-V7 [126,212]. However, this phenomenon can be explained by the context-dependent function of MED12: under some conditions, loss of MED12 eliminates its restraining role toward GLI3, leading to GLI3 hyperactivation, whereas under other conditions MED12 acts as a coactivator of AR/AR-V7 (HYP). These two mechanisms may operate in different subclones or at different stages of the disease, jointly contributing to the formation of the CRPC phenotype (HYP). In addition, it can be hypothesized that binding of MED12 to CDK8/19 reduces the availability of MED12 for participation in the complex that restrains GLI3, thereby functionally liberating the GLI3/AR-V7 pathway (HYP). However, at present there is no direct experimental evidence for either of these hypotheses.

In another study using the VCaP cell line, CDK8/19 was shown to regulate the G1/S transition: selective CDK8/19 inhibitors caused premature G1/S progression coupled with activation of ATR-dependent cell death pathways, pointing to replicative stress as the primary mechanism of cytotoxicity. Notably, the data from [213] directly contradict the results of [10] and [11] regarding the direction of c-MYC regulation upon CDK8/19 inhibition, illustrating a general problem in the field: the specificity and direction of CDK8/19 effects on transcriptional targets appear to be highly context-dependent and cannot yet be considered unequivocally established (HYP).

A broader transcriptomic analysis of transcriptional CDKs in PCa showed that CDK8 acts in coordination with CDK7 and CDK9, sustaining aberrant transcription at all stages of the disease [152,214]. In particular, CDK8/19 support AR-dependent transcriptional programs in CRPC, and inhibition of CDK9, which functions together with CDK8 at the elongation stage, has been reported to enhance anti-tumor effects when combined with androgen-directed treatment [215].

Thus, in PCa, CDK8/19 likely acts simultaneously as a coactivator of AR, sustaining oncogenic transcription at AR-dependent stages, and as a participant in a broader system of transcriptional reprogramming that enables the tumor to adapt to therapeutic pressure (HYP). The combination of direct interaction with the Mediator complex, substrate regulation (STATs, Smads, β-catenin, RNA Pol II), and cell-cycle maintenance makes CDK8/19 a promising target both for monotherapy and for combinatorial therapeutic approaches in PCa.

At the same time, the most compelling functional data on CDK8/19 inhibition described in this section have been obtained in already-established CRPC. These results support the involvement of CDK8/19 in adaptive transcriptional responses, but they do not allow determination of the stage of tumor evolution at which their blockade is most effective. On this basis, particular importance is attached to studying the action of CDK8/19 inhibitors not only in late-stage CRPC models but also in primary tumors of various molecular subtypes and in models of sequential transition from an AR-dependent state to castration resistance. Such an approach would allow for testing of whether blockade of Mediator kinases can disrupt the formation of tumor-specific enhancer complexes and adaptive transcriptional programs before they become firmly established. In this model, the therapeutic potential of CDK8/19 is determined not only by the ability to suppress already-established CRPC but also by the potential capacity to interfere with the process of resistant transcriptional state acquisition (HYP). The advantage of early CDK8/19 inhibition remains a hypothesis and requires direct experimental validation in models of primary PCa and of acquired castration resistance (HYP).

## 4. Critical Assessment of Therapeutic Strategies: From Targeted AR Signaling Suppression to Restriction of Transcriptional Plasticity

### 4.1. Therapeutic Suppression of AR Signaling and the Evolution of Resistance

At early stages of PCa, the tumor generally retains a pronounced dependence on androgen signaling; however, the therapeutic strategy is determined by the disease stage and the risk of progression. In localized PCa, management ranges from active surveillance to radical prostatectomy or radiotherapy; suppression of androgen signaling is used in locally advanced, recurrent, and metastatic disease as monotherapy or as a component of combination treatment [216]. ADT, which reduces circulating androgen levels, has long been the mainstay of systemic therapy for advanced PCa [216].

The recognition of the central role of AR in maintaining tumor cell viability has led to progressively intensified targeting of this signaling pathway. Beyond classical ADT, agents acting at different levels of the AR signaling axis have been introduced into clinical practice: abiraterone reduces androgen synthesis, whereas enzalutamide, apalutamide, and darolutamide directly inhibit AR. Addition of abiraterone, enzalutamide, and apalutamide to ADT significantly improved clinical outcomes in metastatic hormone-sensitive prostate cancer in the LATITUDE, ENZAMET, and TITAN trials, respectively [217,218,219]. For darolutamide, efficacy in metastatic hormone-sensitive PCa was demonstrated both in combination with ADT and docetaxel in the ARASENS trial, which showed an overall survival benefit, and in combination with ADT alone in the ARANOTE trial, which demonstrated a significant improvement in radiographic progression-free survival [220,221]. In castration-resistant disease, the efficacy of AR signaling-suppressing agents has also been confirmed: abiraterone and enzalutamide in mCRPC and apalutamide and darolutamide in high-risk non-metastatic CRPC [222,223,224,225].

### 4.2. Tumor Evolution Under Therapy: Targeting the Resistant State and Mechanisms of State Transition

Under the pressure of ADT and subsequent intensive AR blockade, the tumor cell population undergoes constant selective pressure, promoting the selection and adaptation of cells capable of surviving under conditions of suppressed androgen signaling [6]. At early stages, resistance development predominantly involves preservation of AR dependence and is realized through amplification of the *AR* gene and its regulatory elements, generation of constitutively active variants including AR-V7, and reprogramming of the AR cistrome [3,4]. As further progression occurs, alternative transcriptional programs associated with PI3K/AKT/mTOR, NF-κB, WNT/β-catenin, Hippo/YAP/TAZ, and the MYC family acquire increasing importance, whereas a subset of tumors acquires an AR-low/AR-negative or neuroendocrine phenotype [5,106,113,118,151].

A consequence of such evolution is a change not only in intracellular regulatory networks but also in the available set of therapeutic targets. Therefore, novel clinical strategies can be roughly divided into two fundamentally different classes.

The first class of strategies targets molecular and phenotypic vulnerabilities identified in the already-resistant tumor. These include DNA repair defects, used to select patients for PARP inhibitor therapy, and PSMA expression, enabling PSMA-targeted radioligand therapy [226,227]. A separate direction is represented by surface antigens considered as targets for antibody–drug conjugates. In particular, agents directed against B7-H3 and CD46 are at various stages of clinical development. Ifinatamab deruxtecan is being investigated in mCRPC in the phase I/II IDeate-Prostate02 trial (NCT06863272), and the CD46-targeted FG-3246 in a phase II trial (NCT06842498) [228,229,230]. Another B7-H3-directed antibody–drug conjugate, elfetabarut drozuntentan (BNT324/DB-1311), is being studied in a randomized phase III trial versus docetaxel (NCT07365995) [231].

An important exception to the use of phenotype-directed therapies exclusively in established resistant disease is the expansion of PSMA-targeted radioligand therapy into earlier metastatic settings. PSMA expression is regulated by AR signaling and may increase following AR blockade, potentially enhancing tumor uptake of PSMA-targeted radiopharmaceuticals [232,233]. In the ENZA-p substudy in mCRPC, PSMA PET uptake increased after enzalutamide in 68% of evaluable patients; the interaction between early uptake change and benefit from adding 177Lu-PSMA-617 was suggestive but not statistically conclusive (*p* = 0.055) [233]. The phase III PSMAddition trial demonstrated improved radiographic progression-free survival with luteti-um-177–PSMA-617 combined with ADT and an AR pathway inhibitor (ARPI) in PSMA-positive metastatic hormone-sensitive PCa [234], leading to FDA approval on 31 July 2026 [235].

Thus, as the tumor evolves, the repertoire of therapeutically accessible targets also changes. These strategies allow for targeting of already-existing characteristics of the resistant tumor, but they do not by themselves prevent further shifts in its transcriptional state.

The second class of potential strategies is aimed not so much at the already-established phenotype of the resistant cell but rather at the epigenetic and transcriptional mechanisms that enable its emergence and maintenance. One of the most studied such regulators is EZH2—the catalytic subunit of the PRC2 complex, which mediates trimethylation of H3K27 (H3K27me3) and is involved in repression of genes determining cell differentiation. At the same time, during PCa progression, EZH2 functions are not limited to canonical Polycomb-dependent repression. In CRPC cells, EZH2 can exhibit Polycomb-independent activity and act as a transcriptional coactivator. During PCa progression, EZH2 activity is associated with epigenetic reprogramming and changes in cellular identity, including the emergence of AR-independent and neuroendocrine programs [149,236]. A clinical example of this approach is the use of the selective EZH2 inhibitor mevrometostat (PF-06821497). In combination with enzalutamide, it is being investigated in the randomized phase III MEVPRO-2 trial in mCRPC (NCT06629779) [237]. Tulmimetostat, which inhibits EZH2/EZH1, is also being studied in mCRPC, including in combination with enzalutamide, within the phase I/II trial NCT04104776 [238,239].

These studies do not yet prove the feasibility of clinical prevention of lineage plasticity; however, they show that combining blockade of a dominant oncogenic pathway with targeting of epigenetic state regulators is already a concept being tested in clinical trials.

The differences between the above approaches lie not only in the choice of molecular target but also in the level of intervention in tumor evolution (Table 1). AR-directed therapy suppresses the initial dominant dependency of tumor cells; PARP inhibitors, PSMA-targeted therapy, and antibody–drug conjugates exploit molecular or phenotypic vulnerabilities present in the resistant tumor. In contrast, inhibition of epigenetic and transcriptional state regulators, such as EZH2, implies targeting the mechanisms that maintain or reorganize adaptive transcriptional programs [10,149,236].

### 4.3. CDK8/19 Inhibition as a Potential Barrier to Plasticity

CDK8/19 inhibition is being investigated as a strategy to disrupt adaptive transcription in CRPC. Unlike ADCs, which target tumor-associated surface antigens, CDK8/19 inhibitors act on transcriptional regulation. Whether treatment at earlier stages can delay resistance acquisition or limit lineage plasticity remains to be established.

CDK19 expression is elevated in primary PCa relative to normal prostates, whereas CDK8 expression is lower or largely unchanged at this stage and rises in mCRPC. Increased CDK8 in mCRPC coincides with elevated AR but reduced *KLK3* expression, suggesting altered AR-associated transcription [10]. CDK8/19 inhibition has reduced proliferation or invasion in PCa models [194,211]. Li et al. showed that genetic inactivation or pharmacological inhibition of CDK8/19 restored sensitivity to androgen deprivation and suppressed growth in established CRPC xenografts, including patient-derived models [10].

CDK8 and CDK19 can compensate for each other in supporting tumor growth without necessarily regulating identical genes. In 22Rv1 CRPC xenografts, re-expression of either catalytically active kinase restored castration-resistant growth after double knockout, whereas catalytically inactive variants did not [10]. In VCaP cells, combined depletion of both kinases, but not either alone, impaired proliferation [213]. In a non-prostate model, CDK8 catalytic activity promoted Pol II pause release at a subset of IFN-γ-responsive genes, while CDK19 also showed kinase-independent functions [166]. Comparing CDK8-only and CDK19-only PCa cells by RNA-seq and nascent-transcription assays could reveal whether the two kinases support distinct gene subsets despite their shared effects on growth. Because most tested inhibitors target both kinases, their effects cannot be assigned to either paralog individually.

The next logical step in translational validation of this concept should involve modeling the process of resistance acquisition, rather than merely analyzing already therapy-adapted cellular models. Of particular interest is detailed tracking of the transition from an androgen-dependent state, represented for example by LNCaP, to castration-resistant derivatives such as LNCaP-Abl, in the presence and absence of selective CDK8/19 inhibitors. Additional inclusion of AR-negative PC-3 and DU145 models would allow determination of whether dependence on Mediator kinases is retained after loss of AR signaling. Such a design would enable testing of the key hypothesis: whether early CDK8/19 inhibition can not only suppress already-established CRPC but also restrict the formation of adaptive transcriptional programs that drive resistance development.

Key preclinical studies of CDK8/19 inhibition in prostate cancer models are summarized in Table 2, highlighting both therapeutic opportunities and context-dependent differences in responses to kinase inhibition.

The complementary compound-focused comparison in Table 3 distinguishes the individual dual CDK8/19 inhibitors used in PCa experiments from compounds undergoing clinical investigation in other malignancies; none has demonstrated clinical reversal of ADT/ARPI resistance in PCa.

### 4.4. Combination Strategies and Pharmacological Rationale for Early CDK8/19 Inhibition

Long-term administration of anticancer therapy creates selective pressure that promotes the outgrowth of resistant cell populations [245]. Prolonged androgen receptor blockade with ARPIs (enzalutamide, abiraterone) suppresses AR-dependent growth and can induce regression but simultaneously promotes selection and adaptation of therapy-resistant clones. At the cellular level, this is often accompanied by altered receptor function, which is pathomorphologically registered as mixed nuclear-cytoplasmic staining. Subsequently, surviving mCRPC cells employ transcriptional reprogramming mechanisms, altering surface antigen expression patterns: PSMA expression may decrease in a subset of AR-low/AR-negative or neuroendocrine tumors, despite transient PSMA upregulation following AR pathway blockade in other contexts; alternative surface antigens, including B7-H3 and CD46 [246,247,248,249]. Attempts to use monotherapy with the latest ADCs or degraders (proteolysis-targeting chimeras, PROTACs) at later stages, when antigenic shift has already occurred, yield only transient responses. The tumor rapidly modifies its transcriptome, escaping a single target. Thus, the clinical approach of sequential monotherapy may ultimately be associated with the emergence of new, more aggressive forms of cancer. This justifies a shift toward a new therapeutic concept—from sequential suppression of emerging resistance mechanisms to preemptive targeting of the mechanisms that enable adaptive transcriptional reprogramming of the tumor.

Combined suppression of AR signaling and CDK8/19 activity may enhance anti-tumor efficacy by disrupting adaptive transcriptional programs that enable CRPC cells to survive under androgen-deprived conditions. Preclinical studies support this approach, including enhanced effects in combination-treated prostate cancer cell models and restoration of sensitivity to androgen deprivation with regression of selected CRPC xenografts [10,211].

CDK8/19 inhibition has reduced proliferation, migration, or invasion in PCa models [194,211]. In established CRPC, Li et al. showed that inhibiting CDK8/19 disrupted transcriptional adaptation to androgen deprivation [10].

In AR-negative PC-3 cells, CDK8/19 inhibition reduced migration, and a separate study reported reduced proliferation [11]. In androgen-independent LNCaP-Abl cells, CDK8/19 inhibition substantially reduced viability, while addition of bicalutamide produced a further anti-proliferative effect [211]. These findings support further investigation of combined CDK8/19 and AR inhibition in CRPC.

CDK8/19 inhibition demonstrated anti-tumor activity in established CRPC models, providing a rationale for evaluating combinations with ADT or ARPIs. Earlier intervention might also delay the development of resistance, but this possibility requires direct testing during resistance acquisition [10].

## 5. Discussion

The progression of PCa from an androgen-dependent stage to CRPC remains the major obstacle to successful therapy and the reason for the high mortality rates among PCa patients. Despite the initial efficacy of ARPIs, resistance develops in virtually all patients, driven by the profound biological plasticity of tumor cells. A key mechanism of this adaptation is the reprogramming of the transcriptional apparatus, whereby AR, despite castration-level ligand or even the loss of its full-length isoform AR-FL, preserves or restores its transcriptional activity [3].

A central role in this process is played by the emergence of constitutively active truncated splice variants, among which AR-V7 is the most thoroughly studied. Lacking the ligand-binding domain, AR-V7 retains the N-terminal and DNA-binding domains, allowing it to activate oncogenic programs in the absence of androgens, thereby promoting therapeutic resistance and an adverse prognosis. As emphasized in recent reviews, AR-V7 does not act as an isolated driver but rather as a dynamic hub within a complex adaptive network, interacting with AR-FL through heterodimerization and engaging epigenetic regulators to reprogram transcription [65]. Of particular note is the ability of AR-V7 to initiate transcriptional programs that overlap with, yet are not identical to, those of AR-FL, thus expanding the adaptive potential of tumor cells [4]. Whether these programs are executed through heterodimer formation with AR-FL and/or via AR-V7 homodimerization remains, however, an open question to date. Furthermore, recent data on the possible existence of distinct mechanisms of action for monomeric versus dimeric forms of AR—specifically, their differential effects on c-MYC oncogene activity—have attracted additional interest [146]. Similar patterns of behavior for monomeric and dimeric forms of AR-V7 have not yet been investigated.

Notably, as PCa progresses further, a shift occurs from AR-dependent signaling toward AR-independent mechanisms, including activation of alternative pathways such as PI3K/AKT/mTOR, WNT/β-catenin, NF-κB, and Hippo/YAP/TAZ, as well as deregulation of the MYC family [106,113,119,151]. These signaling networks do not merely bypass AR function but actively shape an aggressive, therapeutically resistant phenotype, making them attractive targets for therapy.

In this context, c-MYC is of particular interest, with *MYC* amplification observed in up to 80% of mCRPC cases according to some reports [127]. The dynamics of the interplay between AR and c-MYC are context-dependent and determine the trajectory of disease progression. In normal prostate cells, AR exerts a suppressive function by repressing c-MYC transcription, whereas upon malignant transformation, AR acquires the ability to activate c-MYC, thereby initiating a proliferative program [139,140]. Moreover, c-MYC overexpression promotes suppression of canonical AR-dependent transcription through redistribution of histone marks and induction of promoter-proximal RNA Pol II pausing at AR target genes, while simultaneously activating its own oncogenic program [143,144]. During the transition to the neuroendocrine phenotype of PCa, a qualitative shift occurs, termed the “MYC family switch,” in which dominant c-MYC is replaced by N-MYC and L-MYC, accompanied by sustained suppression of AR signaling and consolidation of neuroendocrine cell identity [151]. Thus, MYC functions not merely as an oncogene but as a central switch of transcriptional programs that determines tumor phenotypic plasticity. At the same time, the identification of a key role for L-MYC in the tumor’s switch to AR-independent survival mechanisms makes its investigation as a potential therapeutic target in late-stage PCa highly relevant.

It is precisely at the intersection of these two key regulatory axes—AR and MYC—that CDK8/19 reside, as components of the CKM. Unlike classical CDKs, CDK8/19 do not directly control the cell cycle but instead act as systemic integrators of transcriptional signals, modulating RNA Pol II elongation through interactions with the Mediator, SWI/SNF, and SEC [9]. As noted in fundamental reviews, the Mediator kinase module functions as a regulatory node through which changes in gene expression patterns are initiated, and this module is considered an oncogenic unit, wherein pathogenic activation/deactivation via component alterations drives tumorigenesis [250].

Preclinical evidence provides a rationale for targeting CDK8/19 alongside an-drogen-directed therapy. CDK8/19 inhibition enhanced the anti-proliferative effect of enzalutamide in 22Rv1 cells and, with androgen deprivation, induced regression of selected CRPC xenografts in vivo [10,11,171]. CDK8/19 inhibition also reduced proliferation or migration in AR-negative PC-3 cells [211]. Clinical development of CDK8/19-targeting agents remains largely early-stage [251]. Senexin B (BCD-115) was evaluated in a phase I trial in breast cancer [240], whereas RVU120 (SEL120-34A) underwent phase I/II evaluation in advanced solid tumors; the latter trial was terminated by sponsor decision [241]. TSN084 is a multikinase agent in phase I testing outside PCa [243], while SNX631 remains preclinical [10]. Preliminary RVU120 dose-escalation findings describe short-term tolerability and target engagement [242], but clinical efficacy, long-term safety, and reversal of ADT/ARPI resistance in patients with PCa have not been established. Compound-dependent toxicity has also been reported preclinically [252].

However, despite the evident therapeutic potential, the molecular mechanisms underlying the interplay between AR and CDK8/19 remain largely unidentified. Based on the available data, we propose several possible regulatory pathways. First, the effect of CDK8/19 on AR activity may be mediated through regulation of c-MYC- and β-catenin-dependent transcriptional programs [116,187]. Second, another potential mechanism involves regulation of AR expression and ligand-independent activity through phosphorylation of TFs and coregulators associated with STAT3 and NF-κB pathways. Third, CDK8/19 are proposed to participate in shaping AR-, FOXA1-, ERG-, and MYC-dependent transcriptional programs by controlling the recruitment of the SWI/SNF/BAF complex to enhancers [170,204,205]. In the AR context, such chromatin remodeling could alter receptor recruitment to distal regulatory elements and the formation of enhancer–promoter loops.

At the same time, despite their high structural homology, the CDK8 and CDK19 paralogs appear not to be fully functionally equivalent. Thus, outside the context of PCa, it has been shown that CDK8 regulates transcription in response to IFN-γ through its kinase activity, whereas CDK19 mainly functions as a structural scaffold protein [166]. Moreover, in the context of hypoxia, CDK8, but not CDK19, acts as a coactivator of HIF1A target genes [168]. As for PCa, it is known that androgens differentially regulate the expression of these paralogs: they decrease CDK8 and increase CDK19 [193]. However, it currently remains unknown whether the CDK8 and CDK19 paralogs exert differential effects on the activity of AR-FL and its truncated isoforms, such as AR-V7, as well as on the activity of the monomeric and dimeric forms of AR. These questions remain open for further investigation.

Additional complexity in elucidating the regulatory interactions of the CDK8 and CDK19 paralogs arises from the existence of their interchangeable partners within the kinase module—MED12/MED12L and MED13/MED13L—which theoretically allows for the assembly of up to eight distinct module configurations, each potentially possessing unique substrate specificity and tissue distribution. As noted in the review by Clark et al., little is known about the biological or functional role of MED12L and MED13L, and no CDK19-specific kinase targets have been identified in any biological context to date [250]. In PCa, CDK19 expression correlates with progression, Ki67, and AR status; furthermore, CDK19 plays a dominant role in PCa compared with CDK8 [8]. At the same time, the functional interchangeability of the CDK8/19 paralogs in the PCa context remains debatable. Moreover, the role of the alternative kinase module activator—the protein MTBP, which, like MED12, binds CDK8/19 but activates distinct transcriptional programs and interacts with c-MYC through Tip48/Tip49—remains insufficiently studied [198]. In this regard, additional studies are urgently needed to dissect the role of each CDK8/19 paralog, as well as individual kinase module components (MED12, MED13, cyclin C, and MTBP), in the regulation of AR signaling and PCa progression. Of particular interest is the analysis of how different subunit combinations within the module affect transcription of MYC- and AR-related genes and how this knowledge could be leveraged for personalized therapy.

Finally, potential challenges associated with CDK8/19 inhibition cannot be overlooked. First, given that CDK8/19 perform both kinase-dependent and kinase-independent functions (e.g., cyclin C stabilization), selective suppression of catalytic activity alone may be insufficient, and in some contexts, protein degradation may be required. Second, CDK8/19 regulate a wide range of TFs, including STATs, SMAD, β-catenin, and E2F1; therefore, their inhibition may exert unpredictable effects on immune response, cell cycle, and metabolism. Third, some studies report paradoxical effects, such as increased c-MYC levels upon CDK8/19 inhibition in the VCaP cell line [213], necessitating careful analysis of context-dependent responses.

At the same time, achieving high selectivity toward CDK8 and CDK19 over other CDKs and kinases is critical for minimizing off-target effects and toxicity, while the identification of predictive biomarkers for patient selection—those most likely to respond to CDK8/19 inhibition—represents a promising area of research. Finally, long-term CDK8/19 inhibition may lead to activation of compensatory pathways. In particular, given that CDK9, like CDK8/19, is involved in AR- and AR-variant-mediated transcriptional modulation [215], the possibility of acquired resistance to CDK8/19 inhibition requires investigation in longitudinal PCa models.

Thus, CDK8/19 inhibition has produced encouraging results in established CRPC models, including restored sensitivity to castration in selected xenografts [10]. Clinical translation will require published phase II/III studies in ADT/ARPI-resistant CRPC that establish efficacy, combination safety, dose–response relationships, and validated biomarkers. Its ability to delay the acquisition of resistance remains a separate experimental question.

Because CDK8/19 inhibitors act on kinases that modulate multiple transcription factors and may produce context-dependent effects, their clinical use may in the future require identifying potential predictive biomarkers to stratify patients with PCa whose tumors are likely to depend on CDK8/19. In this regard, the relative contributions of CDK8 and CDK19 may change during PCa progression. In AR-V7-positive 22Rv1 xenografts, re-expression of either catalytically active CDK8 or CDK19 restored castration-resistant tumor growth following dual kinase knockout [10]. In non-prostate cells, CDK8 promotes Pol II pause release at a subset of inducible genes, while CDK19 also has kinase-independent functions [166]. Increased CDK8 expression in mCRPC raises the possibility of additional CDK8-dependent gene programs; comparing RNA-seq and nascent transcription in CDK8-only and CDK19-only PCa cells would test this hypothesis.

A testable stratification scheme could combine AR-V7 status with CDK8 expression, using CDK19 as a complementary measure. Four groups—AR-V7-negative/CDK8-high, AR-V7-positive/CDK8-high, AR-V7-negative/CDK8-low, and AR-V7-positive/CDK8-low—could be compared for response to CDK8/19 inhibition. This stratification could establish whether the CDK8/19 dependence demonstrated in AR-V7-positive 22Rv1 cells extends to other PCa models and assess the potential of AR-V7 status and CDK8 expression as predictive biomarkers of response to CDK8/19 inhibition. The prevalence and therapeutic sensitivity of these groups require experimental and clinical validation.

These groups could be evaluated in patient-derived organoids and xenografts, comparing CDK8/19 inhibition alone and in combination with ADT or ARPIs. Separate CDK8 and CDK19 perturbations coupled to RNA-seq and nascent-transcription or Pol II pausing assays could identify the relevant transcriptional dependencies. RNA-expression signatures may ultimately provide clinically accessible markers, but they must first be shown to predict treatment benefit.

## 6. Conclusions

Transcriptional reprogramming contributes to the progression of hormone-sensitive PCa to CRPC and, in some tumors, to AR-independent phenotypes. The CDK8/19 kinase module of the Mediator may play a significant integrating role in this process. CDK8/19 participate in AR-, MYC-, and NF-κB-associated responses, and their inhibition has suppressed growth and restored sensitivity to castration in selected CRPC xenografts. Combination with enzalutamide has also enhanced anti-proliferative activity in PCa cells. These findings support further development of CDK8/19-targeted strategies, including investigation of their potential to delay resistance acquisition. Demonstrating clinical benefit will require phase II/III trials in ADT/ARPI-resistant CRPC, together with validated biomarkers and long-term safety data.

## Figures and Tables

**Table 1:** Levels of therapeutic intervention in the evolution of resistant prostate cancer.

Level of Intervention	Examples	Clinical Maturity	Relationship to Plasticity
Dominant signaling driver	AR signaling pathway inhibitors (ARPIs) [216]	Standard of care	Suppresses the initial AR dependence; simultaneously creates selective pressure
DNA repair vulnerability	PARP inhibitors in HRR/BRCA-deficient tumors [226]	Clinically validated for molecularly selected populations	Exploits a genomic vulnerability present in the tumor population
Surface therapeutic targets	PSMA; B7-H3; CD46 [227,229,230,231]	PSMA—clinically validated; B7-H3/CD46—in clinical development	Targets the molecular phenotype of the resistant tumor
Epigenetic state	EZH2; EZH2/EZH1 [149,236,237,239]	Active clinical investigations	Targets mechanisms maintaining and reorganizing epigenetic state
Transcriptional adaptation	CDK8/19–Mediator [10,11]	Predominantly preclinical data in PCa	Potential restriction of adaptive transcriptional reprogramming

Note: ARPIs—androgen receptor signaling pathway inhibitors; PARP—poly(ADP-ribose) polymerase; HRR—homologous recombination repair; and PSMA—prostate-specific membrane antigen. Clinical status of investigational agents and trials is given as of 21 October 2026.

**Table 2:** Key preclinical findings on CDK8/19 inhibition in prostate cancer models.

Study	Models and Intervention	Main Findings	Limitations
Brägelmann et al., 2017 [194]	LNCaP, PC-3, DU145 and other PCa lines; CDK8/CDK19 siRNA, senexin A	CDK19 or CDK8 knockdown reduced PC-3 migration and invasion; senexin A reduced migration/invasion in responsive lines, with modest proliferation effects.	Predominantly in vitro; individual knockdown did not significantly reduce LNCaP or PC-3 proliferation.
Nakamura et al., 2018 [213]	VCaP cells and VCaP xenografts; T-474/T-418 and genetic perturbation	Inhibition induced premature G1/S transition, *MYC* upregulation, replication stress/ATR-dependent cell death; T-474 inhibited VCaP xenograft growth.	Strong model dependence; results in VCaP do not generalize to all PCa cell lines.
Offermann et al., 2022 [211]	LNCaP, LNCaP-Abl, PC-3, DU145; CDK8/19 inhibitors, bicalutamide	Reduced viability in selected AR-expressing models; enhanced response to bicalutamide in LNCaP/LNCaP-Abl; altered migration in AR-negative cells.	Combination effect was not established in PC-3; mechanism of combination response unresolved.
Li et al., 2024 [10]	Castration-resistant cell-derived and patient-derived xenografts; genetic and pharmacological CDK8/19 inactivation	CDK8/19 inactivation impaired adaptive transcription and restored sensitivity to androgen deprivation, with regression of selected CRPC xenografts. Re-expression of either catalytically active CDK8 or CDK19 restored castration-resistant xenograft growth after double knockout.	Tested predominantly established resistant disease; prevention of resistance emergence not demonstrated.
Andolfi et al., 2024 [11]	LNCaP, 22Rv1 (AR-V7+), PC-3; SEL120-34A; MED12 knockdown; enzalutamide	Both approaches reduced proliferation; CDK8/19 inhibition decreased PSA secretion and additively reduced 22Rv1 proliferation with enzalutamide.	In vitro; MED12 depletion differs mechanistically from CDK8/19 catalytic inhibition.

Note: PCa—prostate cancer; CRPC—castration-resistant prostate cancer; PSA—prostate-specific antigen; and AR-V7—androgen receptor splice variant 7. Results of pharmacological inhibition and genetic depletion are presented as distinct interventions.

**Table 3:** CDK8/19-targeting compounds: Target profile, prostate cancer evidence, and clinical development.

Compound	CDK8/CDK19 Inhibitory Profile	Model/Evidence	AR/AR-V7 Status	Main Outcome	Combination Treatment/Experimental Condition	Clinical Stage/Indication
Senexin A	Dual CDK8/19; no paralog-specific attribution	LNCaP, PC-3, DU145 (in vitro) [194]	Mixed AR+ and AR−; AR-V7 not evaluated	Reduced migration/invasion in responsive PCa lines	None reported in the cited PCa study	Preclinical
T-474	CDK8 1.6 nM; CDK19 1.9 nM (enzyme IC50)	VCaP cells and xenografts [213]	AR+/ERG+; AR-V7 not assessed	G1/S dysregulation, ATR-linked cell death; reduced VCaP growth	None in reported PCa efficacy experiments	Preclinical
T-418	CDK8 23 nM; CDK19 62 nM (enzyme IC50)	VCaP cells [213]	AR+/ERG+; AR-V7 not assessed	Reduced proliferation and altered cell-cycle progression in VCaP	None reported	Preclinical
CCT251545	Dual CDK8/19 (does not distinguish paralogs)	LNCaP, LNCaP-Abl, PC-3, DU145 [211]	AR+ and AR−; AR-V7 not assessed	Changes in migration; limited basal viability effects in tested lines	No CCT251545-specific combination effect established	Preclinical
G02788177.93-1	Dual CDK8/19 (does not distinguish paralogs)	LNCaP, LNCaP-Abl, PC-3, DU145 [211]	AR+ and AR−; AR-V7 not assessed	Reduced LNCaP-Abl viability; altered migration and adhesion	Bicalutamide in LNCaP/LNCaP-Abl	Preclinical
Senexin B (=BCD-115)	Dual CDK8/19	LNCaP and other AR+ PCa models [10]	AR+; AR-V7 varied/not stratified	Reduced induction of selected canonical AR targets (e.g., PSA)	Androgen stimulation in vitro [10]	Phase I breast cancer trial completed; not clinically validated in PCa [240]
SNX631	Dual CDK8/19; target-selective genetic controls	22Rv1, C4-2, LNCaP; CRPC xenografts/PDX [10]	22Rv1 AR-V7+; other models AR+	Restored castration response in resistant xenografts; inhibited selected PDX	Castration/degarelix; enzalutamide (C4-2 in vitro)	Preclinical
SNX631-6	Dual CDK8/19; SNX631 analog	MYC-CaP-CR and CRPC PDX [10]	AR/AR-V7 not systematically stratified	Suppressed MYC-CaP-CR and selected PDX tumor growth	Castration compared, with effects also without castration	Preclinical
RVU120 (=SEL120-34A)	Dual CDK8/19; CDK8 4.4 nM; CDK19 10.4 nM (enzyme IC50)	LNCaP, 22Rv1, PC-3 (in vitro) [11]	LNCaP AR+; 22Rv1 AR-V7+; PC-3 AR−	Reduced proliferation; additive benefit with enzalutamide in 22Rv1	Enzalutamide (22Rv1)	Phase I/II outside PCa; solid-tumor trial terminated (sponsor decision) [241,242]
TSN084	Multikinase agent incl. CDK8/19; also MET, FLT3, TRK	No prostate-specific efficacy established in cited studies	Not applicable/not reported	PCa therapeutic efficacy not established	No PCa combination data	Phase I advanced malignancies, NCT05300438 [243]
E966-0530-45418	Preferential CDK8 inhibition (IC50 = 129 nM); partial CDK19 inhibition in kinase profiling	PC-3 and DU145 cells; PC-3 xenograft [244]	AR-negative models; AR-V7 not assessed	Reduced migration and TGF-β1-associated EMT; decreased invasion-related markers in vivo, with limited cytotoxicity in tested conditions	Experimental condition: TGF-β1 stimulation; no therapeutic combination	Preclinical

Note: IC50 values refer to biochemical assays. AR/AR-V7 status is reported where available. PCa—prostate cancer; PDX—patient-derived xenograft; and ATR—ataxia telangiectasia and Rad3-related kinase. Clinical development status: October 2026. Experimental conditions are not therapeutic combinations; preferential CDK8 inhibition does not imply exclusive CDK8 selectivity.

## Data Availability

No new data were created or analyzed in this study. All data discussed in this review are derived from publicly available peer-reviewed literature. The original sources of the reviewed data can be found in the reference list provided in this article.

## Abbreviations

The following abbreviations are used in this manuscript:

PCa	Prostate cancer
ADT	Androgen-deprivation therapy
CRPC	Castration-resistant prostate cancer
NEPC	Neuroendocrine prostate cancer
AR	Androgen receptor
CDK	Cyclin-dependent kinase
N-PCa	Non-PCa models
HYP	Hypotheses
TF	Transcription factor
NTD	N-terminal domain
AF-1	Activation function-1
DBD	DNA-binding domain
ARE	Androgen-response element
LBD	Ligand-binding domain
HD	Hinge region
NLS	Nuclear localization signal
hARE	Half site androgen-response element
ChIP-seq	Chromatin ImmunoPrecipitation sequencing
SWI/SNF	SWItch/Sucrose Non-Fermentable
PSA	prostate-specific antigen
CARNs	castration-resistant cells expressing NKX3.1
mCRPC	metastatic castration-resistant prostate cancer
LiT	lost in tumor
GiT	gained in tumor
LiM	lost in metastases
GiM	gained in metastases
ctDNA	circulating tumor DNA
AAWS	Antiandrogen Withdrawal Syndrome
GR	glucocorticoid receptor
AR-V	androgen receptor variants
CTC	circulating tumor cell
CE3	cryptic exon 3
AR-FL	full-length AR
Ser	serine
RB	retinoblastoma protein
RNA Pol II	RNA polymerase II
SPA	supraphysiological androgen doses
cMED	core Mediator
PIC	preinitiation complex
CKM	CDK8/19 kinase module
CycC	cyclin C
MTBP	MDM2 binding protein
CTD	C-terminal domain
IDR	intrinsically disordered region
PEC	paused elongation complex
P-TEFb	positive transcription elongation factor b
MEF	mouse embryonic fibroblast
SEC	super elongation complex
GRO-seq	Global Run-On sequencing
PRO-seq	Precision Run-On sequencing
rSmad	receptor-activated Smad protein
ADC	antibody–drug conjugate
ARPIs	androgen receptor signaling inhibitors
PROTAC	proteolysis-targeting chimera

## References

[1] Raychaudhuri R., Lin D.W., Montgomery R.B. (2025). Prostate Cancer: A Review. JAMA.

[2] Cole R.N., Fang Q., Matsuoka K., Wang Z. (2024). Androgen Receptor Inhibitors in Treating Prostate Cancer. Asian J. Androl..

[3] Quistini A., Chierigo F., Fallara G., Depalma M., Tozzi M., Maggi M., Jannello L.M.I., Pellegrino F., Mantica G., Terracciano D. (2025). Androgen Receptor Signalling in Prostate Cancer: Mechanisms of Resistance to Endocrine Therapies. Res. Rep. Urol..

[4] Miller K.J., Henry I., Maylin Z., Smith C., Arunachalam E., Pandha H., Asim M. (2023). A Compendium of Androgen Receptor Variant 7 Target Genes and Their Role in Castration Resistant Prostate Cancer. Front. Oncol..

[5] Giunta E.F., Schepisi G., Bleve S., Serra R., Brighi N., Torresan I., Tassinari E., Zanuccoli S., Lolli C. (2026). Neuroendocrine Prostate Cancer (NEPC) in Focus: State of the Art and Future Prospectives. Discov. Oncol..

[6] Imamura J., Ganguly S., Muskara A., Liao R.S., Nguyen J.K., Weight C., Wee C.E., Gupta S., Mian O.Y. (2023). Lineage Plasticity and Treatment Resistance in Prostate Cancer: The Intersection of Genetics, Epigenetics, and Evolution. Front. Endocrinol..

[7] Dannappel M.V., Sooraj D., Loh J.J., Firestein R. (2019). Molecular and in vivo Functions of the CDK8 and CDK19 Kinase Modules. Front. Cell Dev. Biol..

[8] Becker F., Joerg V., Hupe M.C., Roth D., Krupar R., Lubczyk V., Kuefer R., Sailer V., Duensing S., Kirfel J. (2020). Increased Mediator Complex Subunit CDK19 Expression Associates with Aggressive Prostate Cancer. Int. J. Cancer.

[9] Chen M., Li J., Zhang L., Wang L., Cheng C., Ji H., Altilia S., Ding X., Cai G., Altomare D. (2023). CDK8 and CDK19: Positive Regulators of Signal-Induced Transcription and Negative Regulators of Mediator Complex Proteins. Nucleic Acids Res..

[10] Li J., Hilimire T.A., Liu Y., Wang L., Liang J., Gyorffy B., Sikirzhytski V., Ji H., Zhang L., Cheng C. (2024). Mediator Kinase Inhibition Reverses Castration Resistance of Advanced Prostate Cancer. J. Clin. Investig..

[11] Andolfi C., Bartolini C., Morales E., Gündoğdu B., Puhr M., Guzman J., Wach S., Taubert H., Aigner A., Eder I.E. (2024). MED12 and CDK8/19 Modulate Androgen Receptor Activity and Enzalutamide Response in Prostate Cancer. Endocrinology.

[12] Chen Y., Lan T. (2024). N-Terminal Domain of Androgen Receptor Is a Major Therapeutic Barrier and Potential Pharmacological Target for Treating Castration Resistant Prostate Cancer: A Comprehensive Review. Front. Pharmacol..

[13] Lee X.Y., Van Eynde W., Helsen C., Willems H., Peperstraete K., De Block S., Voet A., Claessens F. (2024). Structural Mechanism Underlying Variations in DNA Binding by the Androgen Receptor. J. Steroid Biochem. Mol. Biol..

[14] Nadal M., Prekovic S., Gallastegui N., Helsen C., Abella M., Zielinska K., Gay M., Vilaseca M., Taulès M., Houtsmuller A.B. (2017). Structure of the Homodimeric Androgen Receptor Ligand-Binding Domain. Nat. Commun..

[15] Johnson M.J., Wasmuth E.V. (2024). Structural Perspectives on the Androgen Receptor, the Elusive Shape-Shifter. Steroids.

[16] Cutress M.L., Whitaker H.C., Mills I.G., Stewart M., Neal D.E. (2008). Structural Basis for the Nuclear Import of the Human Androgen Receptor. J. Cell Sci..

[17] Eftekharzadeh B., Banduseela V.C., Chiesa G., Martínez-Cristóbal P., Rauch J.N., Nath S.R., Schwarz D.M.C., Shao H., Marin-Argany M., Di Sanza C. (2019). Hsp70 and Hsp40 Inhibit an Inter-Domain Interaction Necessary for Transcriptional Activity in the Androgen Receptor. Nat. Commun..

[18] Yu X., Yi P., Hamilton R.A., Shen H., Chen M., Foulds C.E., Mancini M.A., Ludtke S.J., Wang Z., O’Malley B. (2020). Structural Insights of Transcriptionally Active, Full-Length Androgen Receptor Coactivator Complexes. Mol. Cell.

[19] Horie-Inoue K., Bono H., Okazaki Y., Inoue S. (2004). Identification and Functional Analysis of Consensus Androgen Response Elements in Human Prostate Cancer Cells. Biochem. Biophys. Res. Commun..

[20] Centenera M.M., Harris J.M., Tilley W.D., Butler L.M. (2008). The Contribution of Different Androgen Receptor Domains to Receptor Dimerization and Signaling. Mol. Endocrinol..

[21] Bolton E.C., So A.Y., Chaivorapol C., Haqq C.M., Li H., Yamamoto K.R. (2007). Cell- and Gene-Specific Regulation of Primary Target Genes by the Androgen Receptor. Genes Dev..

[22] Yavuz K., Lack N.A. (2025). Coregulators Determine Androgen Receptor Activity in Prostate Cancer. Biosci. Rep..

[23] Tewari A.K., Yardimci G.G., Shibata Y., Sheffield N.C., Song L., Taylor B.S., Georgiev S.G., Coetzee G.A., Ohler U., Furey T.S. (2012). Chromatin Accessibility Reveals Insights into Androgen Receptor Activation and Transcriptional Specificity. Genome Biol..

[24] Chukhu M., Dahiya U.R., Heemers H.V. (2025). Evolving Roles for the Androgen Receptor and Its Protein Interactome in Castration-Resistant Prostate Cancer. Oncogene.

[25] Senapati D., Kumari S., Heemers H.V. (2020). Androgen Receptor Co-Regulation in Prostate Cancer. Asian J. Urol..

[26] Huang C.-C.F., Lingadahalli S., Morova T., Ozturan D., Hu E., Yu I.P.L., Linder S., Hoogstraat M., Stelloo S., Sar F. (2021). Functional Mapping of Androgen Receptor Enhancer Activity. Genome Biol..

[27] Keo V., Lu X., Brea L., Shi X., Yu J., Zhao J.C. (2025). A Hierarchy of Luminal Transcription Factors Defines AR Cistrome and Is Lost in Neuroendocrine Prostate Cancer. Cancer Heterog. Plast..

[28] Jin H.-J., Kim J., Yu J. (2013). Androgen Receptor Genomic Regulation. Transl. Androl. Urol..

[29] Hankey W., Chen Z., Wang Q. (2020). Shaping Chromatin States in Prostate Cancer by Pioneer Transcription Factors. Cancer Res..

[30] Gao S., Patten N.D., Cai C. (2025). Alterations in AR-FOXA1 Signaling in Prostate Cancer Progression and Therapeutic Resistance. J. Natl. Cancer Cent..

[31] Ersoy-Fazlioglu B., Lingadahalli S., Altintas U.B., Cingoz A., Tekoglu E., Yu I.P.L., Dikbas M., Missaghimamaghani O., Yavuz K., Adomat H. (2025). Distinct Transcription Factor Interactions Drive HOXB13 Activity in Different Stages of Prostate Cancer. Proc. Natl. Acad. Sci. USA.

[32] Ordonez-Rubiano S.C., Strohmier B.P., Sood S., Dykhuizen E.C. (2024). SWI/SNF Chromatin Remodelers in Prostate Cancer Progression. Front. Epigenet. Epigenom..

[33] Liu S., Kumari S., Hu Q., Senapati D., Venkadakrishnan V.B., Wang D., DePriest A.D., Schlanger S.E., Ben-Salem S., Valenzuela M.M. (2017). A Comprehensive Analysis of Coregulator Recruitment, Androgen Receptor Function and Gene Expression in Prostate Cancer. eLife.

[34] Nelson P.S., Clegg N., Arnold H., Ferguson C., Bonham M., White J., Hood L., Lin B. (2002). The Program of Androgen-Responsive Genes in Neoplastic Prostate Epithelium. Proc. Natl. Acad. Sci. USA.

[35] Crocetto F., Musone M., Chianese S., Conforti P., Digitale Selvaggio G., Caputo V.F., Falabella R., Del Giudice F., Giulioni C., Cafarelli A. (2025). Blood and Urine-Based Biomarkers in Prostate Cancer: Current Advances, Clinical Applications, and Future Directions. J. Liq. Biopsy.

[36] Gioukaki C., Georgiou A., Gkaralea L.E., Kroupis C., Lazaris A.C., Alamanis C., Thomopoulou G.E. (2023). Unravelling the Role of P300 and TMPRSS2 in Prostate Cancer: A Literature Review. Int. J. Mol. Sci..

[37] Maeda K., Habara M., Kawaguchi M., Matsumoto H., Hanaki S., Masaki T., Sato Y., Matsuyama H., Kunieda K., Nakagawa H. (2022). FKBP51 and FKBP52 Regulate Androgen Receptor Dimerization and Proliferation in Prostate Cancer Cells. Mol. Oncol..

[38] Wang L., Wojcieszak J., Kumar R., Zhao Z., Sun X., Xie S., Winblad B., Pavlov P.F. (2024). FKBP51-Hsp90 Interaction-Deficient Mice Exhibit Altered Endocrine Stress Response and Sex Differences Under High-Fat Diet. Mol. Neurobiol..

[39] Le Magnen C., Virk R.K., Dutta A., Kim J.Y., Panja S., Lopez-Bujanda Z.A., Califano A., Drake C.G., Mitrofanova A., Abate-Shen C. (2018). Cooperation of Loss of NKX3.1 and Inflammation in Prostate Cancer Initiation. Dis. Model. Mech..

[40] Antao A.M., Ramakrishna S., Kim K.-S. (2021). The Role of Nkx3.1 in Cancers and Stemness. Int. J. Stem Cells.

[41] Wilson S., Qi J., Filipp F.V. (2016). Refinement of the Androgen Response Element Based on ChIP-Seq in Androgen-Insensitive and Androgen-Responsive Prostate Cancer Cell Lines. Sci. Rep..

[42] Nash C., Boufaied N., Badescu D., Wang Y.C., Paliouras M., Trifiro M., Ragoussis I., Thomson A.A. (2019). Genome-Wide Analysis of Androgen Receptor Binding and Transcriptomic Analysis in Mesenchymal Subsets during Prostate Development. Dis. Model. Mech..

[43] Severson T., Qiu X., Alshalalfa M., Sjöström M., Quigley D., Bergman A., Long H., Feng F., Freedman M.L., Zwart W. (2022). Androgen Receptor Reprogramming Demarcates Prognostic, Context-Dependent Gene Sets in Primary and Metastatic Prostate Cancer. Clin. Epigenet..

[44] Pomerantz M.M., Li F., Takeda D.Y., Lenci R., Chonkar A., Chabot M., Cejas P., Vazquez F., Cook J., Shivdasani R.A. (2015). The Androgen Receptor Cistrome Is Extensively Reprogrammed in Human Prostate Tumorigenesis. Nat. Genet..

[45] Abeshouse A., Ahn J., Akbani R., Ally A., Amin S., Andry C.D., Annala M., Aprikian A., Armenia J., Arora A. (2015). The Molecular Taxonomy of Primary Prostate Cancer. Cell.

[46] Wasmuth E.V., Hoover E.A., Antar A., Klinge S., Chen Y., Sawyers C.L. (2020). Modulation of Androgen Receptor DNA Binding Activity through Direct Interaction with the ETS Transcription Factor ERG. Proc. Natl. Acad. Sci. USA.

[47] Waltering K.K., Urbanucci A., Visakorpi T. (2012). Androgen Receptor (AR) Aberrations in Castration-Resistant Prostate Cancer. Mol. Cell. Endocrinol..

[48] Khadka S., Jeon H.-Y., Hussain A., Qi J. (2026). Regulation of Androgen Receptor Expression by Enhancer Elements in Prostate Cancer. Exp. Mol. Med..

[49] Zengin Z.B., Henderson N.C., Park J.J., Ali A., Nguyen C., Hwang C., Barata P.C., Bilen M.A., Graham L., Mo G. (2025). Clinical Implications of AR Alterations in Advanced Prostate Cancer: A Multi-Institutional Collaboration. Prostate Cancer Prostatic Dis..

[50] Chen E.J., Sowalsky A.G., Gao S., Cai C., Voznesensky O., Schaefer R., Loda M., True L.D., Ye H., Troncoso P. (2015). Abiraterone Treatment in Castration-Resistant Prostate Cancer Selects for Progesterone Responsive Mutant Androgen Receptors. Clin. Cancer Res..

[51] Romanel A., Tandefelt D.G., Conteduca V., Jayaram A., Casiraghi N., Wetterskog D., Salvi S., Amadori D., Zafeiriou Z., Rescigno P. (2015). Plasma *AR* and Abiraterone-Resistant Prostate Cancer. Sci. Transl. Med..

[52] Joseph J.D., Lu N., Qian J., Sensintaffar J., Shao G., Brigham D., Moon M., Maneval E.C., Chen I., Darimont B. (2013). A Clinically Relevant Androgen Receptor Mutation Confers Resistance to Second-Generation Antiandrogens Enzalutamide and ARN-509. Cancer Discov..

[53] Balbas M.D., Evans M.J., Hosfield D.J., Wongvipat J., Arora V.K., Watson P.A., Chen Y., Greene G.L., Shen Y., Sawyers C.L. (2013). Overcoming Mutation-Based Resistance to Antiandrogens with Rational Drug Design. eLife.

[54] Rathkopf D.E., Smith M.R., Ryan C.J., Berry W.R., Shore N.D., Liu G., Higano C.S., Alumkal J.J., Hauke R., Tutrone R.F. (2017). Androgen Receptor Mutations in Patients with Castration-Resistant Prostate Cancer Treated with Apalutamide. Ann. Oncol..

[55] Leone G., Tucci M., Buttigliero C., Zichi C., Pignataro D., Bironzo P., Vignani F., Scagliotti G.V., Di Maio M. (2018). Antiandrogen Withdrawal Syndrome (AAWS) in the Treatment of Patients with Prostate Cancer. Endocr. Relat. Cancer.

[56] Kuasne H., Rodrigues I.S., Fuganti P.E., Losi-Guembarovski R., Ito K., Kishima M.O., de Rodrigues M.A.F., Rogatto S.R., dos Santos R.M., de Cólus I.M.S. (2010). Polymorphisms in the AR and PSA Genes as Markers of Susceptibility and Aggressiveness in Prostate Cancer. Cancer Investig..

[57] Hatcher D., Daniels G., Osman I., Lee P. (2009). Molecular Mechanisms Involving Prostate Cancer Racial Disparity. Am. J. Transl. Res..

[58] Lange E.M., Sarma A.V., Ray A., Wang Y., Ho L.A., Anderson S.A., Cunningham J.M., Cooney K.A. (2008). The Androgen Receptor CAG and GGN Repeat Polymorphisms and Prostate Cancer Susceptibility in African-American Men: Results from the Flint Men’s Health Study. J. Hum. Genet..

[59] Bishop J.L., Thaper D., Zoubeidi A. (2014). The Multifaceted Roles of STAT3 Signaling in the Progression of Prostate Cancer. Cancers.

[60] Sadrkhanloo M., Paskeh M.D.A., Hashemi M., Raesi R., Motahhary M., Saghari S., Sharifi L., Bokaie S., Mirzaei S., Entezari M. (2023). STAT3 Signaling in Prostate Cancer Progression and Therapy Resistance: An Oncogenic Pathway with Diverse Functions. Biomed. Pharmacother..

[61] Hua Y., Azeem W., Shen Y., Zhang S., Olsen J.R., Øyan A.M., Ke X., Zhang W., Kalland K. (2018). Dual Androgen Receptor (AR) and STAT3 Inhibition by a Compound Targeting the AR Amino-terminal Domain. Pharmacol. Res. Perspect..

[62] Duprat F., Robles C., Castillo M.P., Rivas Y., Mondaca M., Jara N., Roa F., Bertinat R., Toledo J., Paz C. (2023). LOX-1 Activation by oxLDL Induces AR and AR-V7 Expression via NF-κB and STAT3 Signaling Pathways Reducing Enzalutamide Cytotoxic Effects. Int. J. Mol. Sci..

[63] Craft N., Shostak Y., Carey M., Sawyers C.L. (1999). A Mechanism for Hormone-Independent Prostate Cancer through Modulation of Androgen Receptor Signaling by the HER-2/Neu Tyrosine Kinase. Nat. Med..

[64] Wen Y., Hu M.C., Makino K., Spohn B., Bartholomeusz G., Yan D.H., Hung M.C. (2000). HER-2/Neu Promotes Androgen-Independent Survival and Growth of Prostate Cancer Cells through the Akt Pathway. Cancer Res..

[65] Li C., Cheng D., Li P. (2025). Androgen Receptor Dynamics in Prostate Cancer: From Disease Progression to Treatment Resistance. Front. Oncol..

[66] Ye J., Xie Y., Wang J., Wu R., Li D., Yoo K.H., Wusiman D., Cho W.C., Lyu Z., Feng D. (2026). Intratumoral Androgens and Genetic Variants Driving Therapy Resistance in Prostate Cancer. Research.

[67] Sharp A., Coleman I., Yuan W., Sprenger C., Dolling D., Rodrigues D.N., Russo J.W., Figueiredo I., Bertan C., Seed G. (2018). Androgen Receptor Splice Variant-7 Expression Emerges with Castration Resistance in Prostate Cancer. J. Clin. Investig..

[68] Antonarakis E.S., Lu C., Wang H., Luber B., Nakazawa M., Roeser J.C., Chen Y., Mohammad T.A., Chen Y., Fedor H.L. (2014). AR-V7 and Resistance to Enzalutamide and Abiraterone in Prostate Cancer. N. Engl. J. Med..

[69] Armstrong A.J., Halabi S., Luo J., Nanus D.M., Giannakakou P., Szmulewitz R.Z., Danila D.C., Healy P., Anand M., Rothwell C.J. (2019). Prospective Multicenter Validation of Androgen Receptor Splice Variant 7 and Hormone Therapy Resistance in High-Risk Castration-Resistant Prostate Cancer: The PROPHECY Study. J. Clin. Oncol..

[70] Hu R., Dunn T.A., Wei S., Isharwal S., Veltri R.W., Humphreys E., Han M., Partin A.W., Vessella R.L., Isaacs W.B. (2009). Ligand-Independent Androgen Receptor Variants Derived from Splicing of Cryptic Exons Signify Hormone Refractory Prostate Cancer. Cancer Res..

[71] Guo Z., Yang X., Sun F., Jiang R., Linn D.E., Chen H., Chen H., Kong X., Melamed J., Tepper C.G. (2009). A Novel Androgen Receptor Splice Variant Is Upregulated during Prostate Cancer Progression and Promotes Androgen-Depletion-Resistant Growth. Cancer Res..

[72] Kim S., Au C.C., Jamalruddin M.A.B., Abou-Ghali N.E., Mukhtar E., Portella L., Berger A., Worroll D., Vatsa P., Rickman D.S. (2022). AR-V7 Exhibits Non-Canonical Mechanisms of Nuclear Import and Chromatin Engagement in Castrate-Resistant Prostate Cancer. eLife.

[73] Charoensawan V., Martinho C., Wigge P.A. (2015). “Hit-and-Run”: Transcription Factors Get Caught in the Act. BioEssays.

[74] Shah M., Funnell A.P.W., Quinlan K.G.R., Crossley M. (2019). Hit and Run Transcriptional Repressors Are Difficult to Catch in the Act. BioEssays.

[75] Roggero C.M., Jin L., Cao S., Sonavane R., Kopplin N.G., Ta H., Ekoue D.N., Witwer M., Ma S., Liu H. (2021). A Detailed Characterization of Stepwise Activation of the Androgen Receptor Variant 7 in Prostate Cancer Cells. Oncogene.

[76] Cai L., Tsai Y.-H., Wang P., Wang J., Li D., Fan H., Zhao Y., Bareja R., Lu R., Wilson E.M. (2018). ZFX Mediates Non-Canonical Oncogenic Functions of the Androgen Receptor Splice Variant 7 in Castrate-Resistant Prostate Cancer. Mol. Cell.

[77] Kim E.H., Cao D., Mahajan N.P., Andriole G.L., Mahajan K. (2020). ACK1–AR and AR–HOXB13 Signaling Axes: Epigenetic Regulation of Lethal Prostate Cancers. NAR Cancer.

[78] Chen Z., Wu D., Thomas-Ahner J.M., Lu C., Zhao P., Zhang Q., Geraghty C., Yan P.S., Hankey W., Sunkel B. (2018). Diverse AR-V7 Cistromes in Castration-Resistant Prostate Cancer Are Governed by HoxB13. Proc. Natl. Acad. Sci. USA.

[79] Cato L., de Tribolet-Hardy J., Lee I., Rottenberg J.T., Coleman I., Melchers D., Houtman R., Xiao T., Li W., Uo T. (2019). ARv7 Represses Tumor-Suppressor Genes in Castration-Resistant Prostate Cancer. Cancer Cell.

[80] Abida W., Cyrta J., Heller G., Prandi D., Armenia J., Coleman I., Cieslik M., Benelli M., Robinson D., Van Allen E.M. (2019). Genomic Correlates of Clinical Outcome in Advanced Prostate Cancer. Proc. Natl. Acad. Sci. USA.

[81] Ma M., Zhu Y., Xiao C., Li R., Cao X., Kang R., Wang X., Li E. (2024). Novel Insights into RB1 in Prostate Cancer Lineage Plasticity and Drug Resistance. Tumori.

[82] Maylin Z.R., Smith C., Classen A., Asim M., Pandha H., Wang Y. (2024). Therapeutic Exploitation of Neuroendocrine Transdifferentiation Drivers in Prostate Cancer. Cells.

[83] Mu P., Zhang Z., Benelli M., Karthaus W.R., Hoover E., Chen C.-C., Wongvipat J., Ku S.-Y., Gao D., Cao Z. (2017). SOX2 Promotes Lineage Plasticity and Antiandrogen Resistance in TP53- and RB1-Deficient Prostate Cancer. Science.

[84] Ku S.Y., Rosario S., Wang Y., Mu P., Seshadri M., Goodrich Z.W., Goodrich M.M., Labbé D.P., Gomez E.C., Wang J. (2017). Rb1 and Trp53 Cooperate to Suppress Prostate Cancer Lineage Plasticity, Metastasis, and Antiandrogen Resistance. Science.

[85] Kareta M.S., Gorges L.L., Hafeez S., Benayoun B.A., Marro S., Zmoos A.-F., Cecchini M.J., Spacek D., Batista L.F.Z., O’Brien M. (2015). Inhibition of Pluripotency Networks by the Rb Tumor Suppressor Restricts Reprogramming and Tumorigenesis. Cell Stem Cell.

[86] Dick F.A., Goodrich D.W., Sage J., Dyson N.J. (2018). Non-Canonical Functions of the RB Protein in Cancer. Nat. Rev. Cancer.

[87] Li H., Wang L., Li Z., Geng X., Li M., Tang Q., Wu C., Lu Z. (2020). SOX2 Has Dual Functions as a Regulator in the Progression of Neuroendocrine Prostate Cancer. Lab. Investig. J. Tech. Methods Pathol..

[88] Keo V., Lu X., Zhao J.C., Yu J. (2026). Genetic and Epigenetic Mechanisms Underlying Treatment-Induced Neuroendocrine Prostate Cancer. J. Natl. Cancer Cent..

[89] Esfini Farahani M., Zhang Y., Akinyemi A.O., Seilani F., Alam M.R., Liu X. (2025). Unlocking the Role of OCT4 in Cancer Lineage Plasticity: A Cross-Cancer Perspective with an Emphasis on Prostate Cancer. Biomedicines.

[90] Guo C., Kadier A., Zhang Z., Mao S., Yang B., Zheng J., Yao X. (2025). ADT Increases Prostate Cancer Cell Invasion via Altering AR/SALL4/SOX2-OCT4 Stem Cell Signaling. Cell Biol. Toxicol..

[91] Kim D.-H., Sun D., Storck W.K., Welker Leng K., Jenkins C., Coleman D.J., Sampson D., Guan X., Kumaraswamy A., Rodansky E.S. (2021). BET Bromodomain Inhibition Blocks an AR-Repressed, E2F1-Activated Treatment-Emergent Neuroendocrine Prostate Cancer Lineage Plasticity Program. Clin. Cancer Res..

[92] Kim Y.-R., Oh K.-J., Park R.-Y., Xuan N.T., Kang T.-W., Kwon D.-D., Choi C., Kim M.S., Nam K.I., Ahn K.Y. (2010). HOXB13 Promotes Androgen Independent Growth of LNCaP Prostate Cancer Cells by the Activation of E2F Signaling. Mol. Cancer.

[93] Kim Y.-R., Kim I.-J., Kang T.W., Choi C., Kim K.K., Kim M.S., Nam K.I., Jung C. (2014). HOXB13 Downregulates Intracellular Zinc and Increases NF-κB Signaling to Promote Prostate Cancer Metastasis. Oncogene.

[94] Bhanvadia R.R., VanOpstall C., Brechka H., Barashi N.S., Gillard M., McAuley E.M., Vasquez J.M., Paner G., Chan W.-C., Andrade J. (2018). MEIS1 and MEIS2 Expression and Prostate Cancer Progression: A Role For HOXB13 Binding Partners in Metastatic Disease. Clin. Cancer Res..

[95] VanOpstall C., Perike S., Brechka H., Gillard M., Lamperis S., Zhu B., Brown R., Bhanvadia R., Vander Griend D.J. (2020). MEIS-Mediated Suppression of Human Prostate Cancer Growth and Metastasis through HOXB13-Dependent Regulation of Proteoglycans. eLife.

[96] Patel R.A., Sayar E., Coleman I., Roudier M.P., Hanratty B., Low J.-Y., Jaiswal N., Ajkunic A., Dumpit R., Ercan C. (2024). Characterization of HOXB13 expression patterns in localized and metastatic castration resistant prostate cancer. J. Pathol..

[97] Cheng S., Yang S., Shi Y., Shi R., Yeh Y., Yu X. (2021). Neuroendocrine prostate cancer has distinctive, non-prostatic HOX code that is represented by the loss of HOXB13 expression. Sci. Rep..

[98] Tang F., Xu D., Wang S., Wong C.K., Martinez-Fundichely A., Lee C.J., Cohen S., Park J., Hill C.E., Eng K. (2022). Chromatin Profiles Classify Castration-Resistant Prostate Cancers Suggesting Therapeutic Targets. Science.

[99] Krishna M., Garg P., Horne D., Salgia R., Singhal S.S. (2026). Inflammation-Driven Transcriptional Reprogramming in Prostate Cancer: Convergence of NFκB, JAK/STAT3, and Epigenetic Remodeling in Castration-Resistant Disease. Cancer Lett..

[100] Zhang L., Altuwaijri S., Deng F., Chen L., Lal P., Bhanot U.K., Korets R., Wenske S., Lilja H.G., Chang C. (2009). NF-κB Regulates Androgen Receptor Expression and Prostate Cancer Growth. Am. J. Pathol..

[101] Jin R., Yamashita H., Yu X., Wang J., Franco O.E., Wang Y., Hayward S.W., Matusik R.J. (2015). Inhibition of NF-Kappa B Signaling Restores Responsiveness of Castrate Resistant Prostate Cancer Cells to Anti-Androgen Treatment by Decreasing Androgen Receptor Variants Expression. Oncogene.

[102] Austin D.C., Strand D.W., Love H.L., Franco O.E., Jang A., Grabowska M.M., Miller N.L., Hameed O., Clark P.E., Fowke J.H. (2016). NF-κB and Androgen Receptor Variant Expression Correlate with Human BPH Progression. Prostate.

[103] Morel K.L., Hamid A.A., Clohessy J.G., Pandell N., Ellis L., Sweeney C.J. (2021). NF-κB Blockade with Oral Administration of Dimethylaminoparthenolide (DMAPT), Delays Prostate Cancer Resistance to Androgen Receptor (AR) Inhibition and Inhibits AR Variants. Mol. Cancer Res. MCR.

[104] Chang K.-S., Chen S.-T., Sung H.-C., Hsu S.-Y., Lin W.-Y., Hou C.-P., Lin Y.-H., Feng T.-H., Tsui K.-H., Juang H.-H. (2023). Androgen Receptor Upregulates Mucosa-Associated Lymphoid Tissue 1 to Induce NF-κB Activity via Androgen-Dependent and -Independent Pathways in Prostate Carcinoma Cells. Int. J. Mol. Sci..

[105] Malinen M., Niskanen E.A., Kaikkonen M.U., Palvimo J.J. (2017). Crosstalk between Androgen and Pro-Inflammatory Signaling Remodels Androgen Receptor and NF-κB Cistrome to Reprogram the Prostate Cancer Cell Transcriptome. Nucleic Acids Res..

[106] Basílio J., Hochreiter B., Hoesel B., Sheshori E., Mussbacher M., Hanel R., Schmid J.A. (2022). Antagonistic Functions of Androgen Receptor and NF-κB in Prostate Cancer—Experimental and Computational Analyses. Cancers.

[107] Nelius T., Filleur S., Yemelyanov A., Budunova I., Shroff E., Mirochnik Y., Aurora A., Veliceasa D., Xiao W., Wang Z. (2007). Androgen Receptor Targets NFjB and TSP1 to Suppress Prostate Tumor Growth in vivo. Int. J. Cancer.

[108] Jamaspishvili T., Berman D.M., Ross A.E., Scher H.I., De Marzo A.M., Squire J.A., Lotan T.L. (2018). Clinical Implications of PTEN Loss in Prostate Cancer. Nat. Rev. Urol..

[109] Nidai Ozes O., Mayo L.D., Gustin J.A., Pfeffer S.R., Pfeffer L.M., Donner D.B. (1999). NF-κB Activation by Tumour Necrosis Factor Requires the Akt Serine–Threonine Kinase. Nature.

[110] Mulholland D.J., Tran L.M., Li Y., Cai H., Morim A., Wang S., Plaisier S., Garraway I.P., Huang J., Graeber T.G. (2011). Cell Autonomous Role of PTEN in Regulating Castration-Resistant Prostate Cancer Growth. Cancer Cell.

[111] Carver B.S., Chapinski C., Wongvipat J., Hieronymus H., Chen Y., Chandarlapaty S., Arora V.K., Le C., Koutcher J., Scher H. (2011). Reciprocal Feedback Regulation of PI3K and Androgen Receptor Signaling in PTEN-Deficient Prostate Cancer. Cancer Cell.

[112] Han X., Zhang A., Wang P., Bi H., Ren K., Li E., Yang X., Aydemir I., Tao K., Lin J. (2024). Pleckstrin-2 Mediates the Activation of AKT in Prostate Cancer and Is Repressed by Androgen Receptor. Am. J. Pathol..

[113] Shorning B.Y., Dass M.S., Smalley M.J., Pearson H.B. (2020). The PI3K-AKT-mTOR Pathway and Prostate Cancer: At the Crossroads of AR, MAPK, and WNT Signaling. Int. J. Mol. Sci..

[114] Tortorella E., Giantulli S., Sciarra A., Silvestri I. (2023). AR and PI3K/AKT in Prostate Cancer: A Tale of Two Interconnected Pathways. Int. J. Mol. Sci..

[115] Bowen C., Ostrowski M.C., Leone G., Gelmann E.P. (2019). Loss of PTEN Accelerates NKX3.1 Degradation to Promote Prostate Cancer Progression. Cancer Res..

[116] Wang G., Wang J., Sadar M.D. (2008). Crosstalk between the Androgen Receptor and β-Catenin in Castrate Resistant Prostate Cancer. Cancer Res..

[117] Patel R., Brzezinska E.A., Repiscak P., Ahmad I., Mui E., Gao M., Blomme A., Harle V., Tan E.H., Malviya G. (2020). Activation of β-Catenin Cooperates with Loss of Pten to Drive AR-Independent Castration-Resistant Prostate Cancer. Cancer Res..

[118] Salem O., Hansen C.G. (2019). The Hippo Pathway in Prostate Cancer. Cells.

[119] Dad H., Hansen A. (2024). Advancements in Research on Non-AR- Signaling Pathways and Targeted Therapies for Castration-Resistant Prostate Cancer. Ann. Urol. Oncol..

[120] Azzolin L., Panciera T., Soligo S., Enzo E., Bicciato S., Dupont S., Bresolin S., Frasson C., Basso G., Guzzardo V. (2014). YAP/TAZ Incorporation in the β-Catenin Destruction Complex Orchestrates the Wnt Response. Cell.

[121] Li X., Zhuo S., Cho Y.S., Liu Y., Yang Y., Zhu J., Jiang J. (2023). YAP Antagonizes TEAD-mediated AR Signaling and Prostate Cancer Growth. EMBO J..

[122] Xue C., Chu Q., Shi Q., Zeng Y., Lu J., Li L. (2025). Wnt Signaling Pathways in Biology and Disease: Mechanisms and Therapeutic Advances. Signal Transduct. Target. Ther..

[123] Lo Ré A.E., Fernández-Barrena M.G., Almada L.L., Mills L.D., Elsawa S.F., Lund G., Ropolo A., Molejon M.I., Vaccaro M.I., Fernandez-Zapico M.E. (2012). Novel AKT1-GLI3-VMP1 Pathway Mediates KRAS Oncogene-Induced Autophagy in Cancer Cells. J. Biol. Chem..

[124] Li N., Truong S., Nouri M., Moore J., Al Nakouzi N., Lubik A.A., Buttyan R. (2018). Non-Canonical Activation of Hedgehog in Prostate Cancer Cells Mediated by the Interaction of Transcriptionally Active Androgen Receptor Proteins with Gli3. Oncogene.

[125] Kaushal J.B., Raut P., Halder S., Alsafwani Z.W., Parte S., Sharma G., Abdullah K.M., Seshacharyulu P., Lele S.M., Batra S.K. (2025). Oncogenic Potential of Truncated-Gli3 via the Gsk3β/Gli3/AR-V7 Axis in Castration-Resistant Prostate Cancer. Oncogene.

[126] Nuñez-Olvera S.I., Cortés-Malagón E.M., Montúfar-Robles I., Flores-Estrada J.J., Alvarez-Sánchez M.E., Puente-Rivera J. (2026). The GLI3–Androgen Receptor Axis: A Feedback Circuit Sustaining Shh Signaling in Prostate Cancer. Receptors.

[127] Schoenborn J.R., Nelson P., Fang M. (2013). Genomic Profiling Defines Subtypes of Prostate Cancer with the Potential for Therapeutic Stratification. Clin. Cancer Res..

[128] Bretones G., Delgado M.D., León J. (2015). Myc and Cell Cycle Control. Biochim. Biophys. Acta.

[129] Faskhoudi M.A., Molaei P., Sadrkhanloo M., Orouei S., Hashemi M., Bokaie S., Rashidi M., Entezari M., Zarrabi A., Hushmandi K. (2022). Molecular Landscape of C-Myc Signaling in Prostate Cancer: A Roadmap to Clinical Translation. Pathol. Res. Pract..

[130] Boufaied N., Chetta P., Hallal T., Cacciatore S., Lalli D., Luthold C., Homsy K., Imada E.L., Syamala S., Photopoulos C. (2024). Obesogenic High-Fat Diet and MYC Cooperate to Promote Lactate Accumulation and Tumor Microenvironment Remodeling in Prostate Cancer. Cancer Res..

[131] Duyao M.P., Buckler A.J., Sonenshein G.E. (1990). Interaction of an NF-Kappa B-like Factor with a Site Upstream of the c-Myc Promoter. Proc. Natl. Acad. Sci. USA.

[132] Moser B., Hochreiter B., Basílio J., Gleitsmann V., Panhuber A., Pardo-Garcia A., Hoesel B., Salzmann M., Resch U., Noreen M. (2021). The Inflammatory Kinase IKKα Phosphorylates and Stabilizes C-Myc and Enhances Its Activity. Mol. Cancer.

[133] Nadiminty N., Tummala R., Liu C., Lou W., Evans C.P., Gao A.C. (2015). NF-kappaB2/P52:C-Myc:hnRNPA1 Pathway Regulates Expression of Androgen Receptor Splice Variants and Enzalutamide Sensitivity in Prostate Cancer. Mol. Cancer Ther..

[134] Sears R., Nuckolls F., Haura E., Taya Y., Tamai K., Nevins J.R. (2000). Multiple Ras-Dependent Phosphorylation Pathways Regulate Myc Protein Stability. Genes Dev..

[135] Welcker M., Orian A., Jin J., Grim J.A., Harper J.W., Eisenman R.N., Clurman B.E. (2004). The Fbw7 Tumor Suppressor Regulates Glycogen Synthase Kinase 3 Phosphorylation-Dependent c-Myc Protein Degradation. Proc. Natl. Acad. Sci. USA.

[136] Майкл П., Труитт М.Л., Сиддики И.Н., Дакер Г.С., Шoкат К.М., Руджерo Д. (2013). Myc and mTOR Converge on a Common Node in Protein Synthesis Control That Confers Synthetic Lethality in Myc-Driven Cancers. Proc. Natl. Acad. Sci. USA.

[137] He T.C., Sparks A.B., Rago C., Hermeking H., Zawel L., da Costa L.T., Morin P.J., Vogelstein B., Kinzler K.W. (1998). Identification of C-MYC as a Target of the APC Pathway. Science.

[138] Antony L., van der Schoor F., Dalrymple S.L., Isaacs J.T. (2014). Androgen Receptor (AR) Suppresses Normal Human Prostate Epithelial Cell Proliferation via AR/β-Catenin/TCF-4 Complex Inhibition of c-MYC Transcription. Prostate.

[139] Vander Griend D.J., Litvinov I.V., Isaacs J.T. (2014). Conversion of Androgen Receptor Signaling From a Growth Suppressor in Normal Prostate Epithelial Cells to an Oncogene in Prostate Cancer Cells Involves a Gain of Function in C-Myc Regulation. Int. J. Biol. Sci..

[140] Guo J., Wei Z., Jia T., Wang L., Nama N., Liang J., Liao X., Liu X., Gao Y., Liu X. (2023). Dissecting Transcription of the 8q24-MYC Locus in Prostate Cancer Recognizes the Equilibration between Androgen Receptor Direct and Indirect Dual-Functions. J. Transl. Med..

[141] Guo H., Wu Y., Nouri M., Spisak S., Russo J.W., Sowalsky A.G., Pomerantz M.M., Wei Z., Korthauer K., Seo J.-H. (2021). Androgen Receptor and MYC Equilibration Centralizes on Developmental Super-Enhancer. Nat. Commun..

[142] Bai S., Cao S., Jin L., Kobelski M., Schouest B., Wang X., Ungerleider N., Baddoo M., Zhang W., Corey E. (2019). A Positive Role of C-Myc in Regulating Androgen Receptor and Its Splice Variants in Prostate Cancer. Oncogene.

[143] Barfeld S.J., Urbanucci A., Itkonen H.M., Fazli L., Hicks J.L., Thiede B., Rennie P.S., Yegnasubramanian S., DeMarzo A.M., Mills I.G. (2017). C-Myc Antagonises the Transcriptional Activity of the Androgen Receptor in Prostate Cancer Affecting Key Gene Networks. EBioMedicine.

[144] Qiu X., Boufaied N., Hallal T., Feit A., de Polo A., Luoma A.M., Alahmadi W., Larocque J., Zadra G., Xie Y. (2022). MYC Drives Aggressive Prostate Cancer by Disrupting Transcriptional Pause Release at Androgen Receptor Targets. Nat. Commun..

[145] Sena L.A. (2025). Polyamine Metabolism in Prostate Cancer. Curr. Opin. Oncol..

[146] Safi R., Wardell S.E., Watkinson P., Qin X., Lee M., Park S., Krebs T., Dolan E.L., Blattler A., Tsuji T. (2024). Androgen Receptor Monomers and Dimers Regulate Opposing Biological Processes in Prostate Cancer Cells. Nat. Commun..

[147] Nyquist M.D., Coleman I.M., Lucas J.M., Li D., Hanratty B., Meade H., Mostaghel E.A., Plymate S.R., Corey E., Haffner M.C. (2023). Supraphysiological Androgens Promote the Tumor Suppressive Activity of the Androgen Receptor through cMYC Repression and Recruitment of the DREAM Complex. Cancer Res..

[148] Hylton-McComas H.M., Cordes A., Floros K.V., Faber A., Drapkin B.J., Miles W.O. (2025). Myc Family Proteins: Molecular Drivers of Tumorigenesis and Resistance in Neuroendocrine Tumors. Biochim. Biophys. Acta Rev. Cancer.

[149] Dardenne E., Beltran H., Benelli M., Gayvert K., Berger A., Puca L., Cyrta J., Sboner A., Noorzad Z., MacDonald T. (2016). N-Myc Induces an EZH2-Mediated Transcriptional Program Driving Neuroendocrine Prostate Cancer. Cancer Cell.

[150] Boutros P.C., Fraser M., Harding N.J., de Borja R., Trudel D., Lalonde E., Meng A., Hennings-Yeomans P.H., McPherson A., Sabelnykova V.Y. (2015). Spatial Genomic Heterogeneity within Localized, Multifocal Prostate Cancer. Nat. Genet..

[151] Sivalingam J., Ballagh K., Cho K.H., Shi Y., Li L., Barron P., Lan M.S., Franco O.E., Yu X. (2026). A MYC Family Switch: L-MYC Drives and Maintains Neuroendocrine Lineage Programs in Prostate Cancer. Neoplasia.

[152] Rahman R., Selth L.A. (2025). Cyclin-Dependent Kinases as Mediators of Aberrant Transcription in Prostate Cancer. Transl. Oncol..

[153] Daniels D.L. (2013). Mutual Exclusivity of MED12/MED12L, MED13/13L, and CDK8/19 Paralogs Revealed within the CDK-Mediator Kinase Module. J. Proteom. Bioinform..

[154] Luyties O., Taatjes D.J. (2022). The Mediator Kinase Module: An Interface between Cell Signaling and Transcription. Trends Biochem. Sci..

[155] Zaffar E., Vieyto-Nuñez J., Pravi K., Aslam M.F., Almeida-Hernández Y., Mieres-Pérez J., Kaschani F., Kaiser M., Kroesen A., Boos D. (2025). MTBP Allosterically Activates Cdk8-CycC Kinase Activity. bioRxiv.

[156] Köhler K., Sanchez-Pulido L., Höfer V., Marko A., Ponting C.P., Snijders A.P., Feederle R., Schepers A., Boos D. (2019). The Cdk8/19-Cyclin C Transcription Regulator Functions in Genome Replication through Metazoan Sld7. PLoS Biol..

[157] Galbraith M.D., Andrysik Z., Pandey A., Hoh M., Bonner E.A., Hill A.A., Sullivan K.D., Espinosa J.M. (2017). CDK8 Kinase Activity Promotes Glycolysis. Cell Rep..

[158] Allen B.L., Taatjes D.J. (2015). The Mediator Complex: A Central Integrator of Transcription. Nat. Rev. Mol. Cell Biol..

[159] Rengachari S., Schilbach S., Aibara S., Dienemann C., Cramer P. (2021). Structure of the Human Mediator–RNA Polymerase II Pre-Initiation Complex. Nature.

[160] Abdella R., Talyzina A., Chen S., Inouye C.J., Tjian R., He Y. (2021). Structure of the Human Mediator-Bound Transcription Pre-Initiation Complex. Science.

[161] Zhao H., Li J., Xiang Y., Malik S., Vartak S.V., Veronezi G.M.B., Young N., Ryner M., Kalchschmidt J., Conte A. (2024). An IDR-Dependent Mechanism for Nuclear Receptor Control of Mediator Interaction with RNA Polymerase II. Mol. Cell.

[162] Chao T.-C., Chen S.-F., Kim H.J., Tang H.-C., Tseng H.-C., Xu A., Palao L., Khadka S., Li T., Huang M.-F. (2024). Structural Basis of the Human Transcriptional Mediator Regulated by Its Dissociable Kinase Module. Mol. Cell.

[163] Vos S.M., Farnung L., Urlaub H., Cramer P. (2018). Structure of Paused Transcription Complex Pol II-DSIF-NELF. Nature.

[164] Zhan Y., Grabbe F., Oberbeckmann E., Dienemann C., Cramer P. (2024). Three-Step Mechanism of Promoter Escape by RNA Polymerase II. Mol. Cell.

[165] Takahashi H., Parmely T.J., Sato S., Tomomori-Sato C., Banks C.A.S., Kong S.E., Szutorisz H., Swanson S.K., Martin-Brown S., Washburn M.P. (2011). Human Mediator Subunit MED26 Functions as a Docking Site for Transcription Elongation Factors. Cell.

[166] Steinparzer I., Sedlyarov V., Rubin J.D., Eislmayr K., Galbraith M.D., Levandowski C.B., Vcelkova T., Sneezum L., Wascher F., Amman F. (2019). Transcriptional Responses to IFN-γ Require Mediator Kinase-Dependent Pause Release and Mechanistically Distinct CDK8 and CDK19 Functions. Mol. Cell.

[167] Donner A.J., Ebmeier C.C., Taatjes D.J., Espinosa J.M. (2010). CDK8 Is a Positive Regulator of Transcriptional Elongation within the Serum Response Network. Nat. Struct. Mol. Biol..

[168] Galbraith M.D., Allen M.A., Bensard C.L., Wang X., Schwinn M.K., Qin B., Long H.W., Daniels D.L., Hahn W.C., Dowell R.D. (2013). HIF1A Employs CDK8-Mediator to Stimulate RNAPII Elongation. Cell.

[169] Vos S.M., Farnung L., Boehning M., Wigge C., Linden A., Urlaub H., Cramer P. (2018). Structure of Activated Transcription Complex Pol II–DSIF–PAF–SPT6. Nature.

[170] Dannappel M.V., Zhu D., Sun X., Chua H.K., Poppelaars M., Suehiro M., Khadka S., Lim Kam Sian T.C.C., Sooraj D., Loi M. (2022). CDK8 and CDK19 Regulate Intestinal Differentiation and Homeostasis via the Chromatin Remodeling Complex SWI/SNF. J. Clin. Investig..

[171] Chen M., Liang J., Ji H., Yang Z., Altilia S., Hu B., Schronce A., McDermott M.S.J., Schools G.P., Lim C. (2017). CDK8/19 Mediator Kinases Potentiate Induction of Transcription by NFκB. Proc. Natl. Acad. Sci. USA.

[172] Serrao A., Jenkins L.M., Chumanevich A.A., Horst B., Liang J., Gatza M.L., Lee N.Y., Roninson I.B., Broude E.V., Mythreye K. (2018). Mediator Kinase CDK8/CDK19 Drives YAP1-Dependent BMP4-Induced EMT in Cancer. Oncogene.

[173] Hou Y., Huttenlocher A. (2024). Advancing Chemokine Research: The Molecular Function of CXCL8. J. Clin. Investig..

[174] Yamamoto S., Hagihara T., Horiuchi Y., Okui A., Wani S., Yoshida T., Inoue T., Tanaka A., Ito T., Hirose Y. (2017). Mediator Cyclin-Dependent Kinases Upregulate Transcription of Inflammatory Genes in Cooperation with NF-κB and C/EBPβ on Stimulation of Toll-like Receptor 9. Genes Cells Devoted Mol. Cell. Mech..

[175] Bancerek J., Poss Z.C., Steinparzer I., Sedlyarov V., Pfaffenwimmer T., Mikulic I., Dölken L., Strobl B., Müller M., Taatjes D.J. (2013). CDK8 Kinase Phosphorylates Transcription Factor STAT1 to Selectively Regulate the Interferon Response. Immunity.

[176] Putz E.M., Gotthardt D., Hoermann G., Csiszar A., Wirth S., Berger A., Straka E., Rigler D., Wallner B., Jamieson A.M. (2013). CDK8-Mediated STAT1-S727 Phosphorylation Restrains NK Cell Cytotoxicity and Tumor Surveillance. Cell Rep..

[177] Martinez-Fabregas J., Wang L., Pohler E., Cozzani A., Wilmes S., Kazemian M., Mitra S., Moraga I. (2020). CDK8 Fine-Tunes IL-6 Transcriptional Activities by Limiting STAT3 Resident Time at the Gene Loci. Cell Rep..

[178] Rzymski T., Mikula M., Żyłkiewicz E., Dreas A., Wiklik K., Gołas A., Wójcik K., Masiejczyk M., Wróbel A., Dolata I. (2017). SEL120-34A Is a Novel CDK8 Inhibitor Active in AML Cells with High Levels of Serine Phosphorylation of STAT1 and STAT5 Transactivation Domains. Oncotarget.

[179] Menzl I., Witalisz-Siepracka A., Sexl V. (2019). CDK8-Novel Therapeutic Opportunities. Pharmaceuticals.

[180] Alarcón C., Zaromytidou A.-I., Xi Q., Gao S., Yu J., Fujisawa S., Barlas A., Miller A.N., Manova-Todorova K., Macias M.J. (2009). Nuclear CDKs Drive Smad Transcriptional Activation and Turnover in BMP and TGF-β Pathways. Cell.

[181] Aragón E., Goerner N., Zaromytidou A.-I., Xi Q., Escobedo A., Massagué J., Macias M.J. (2011). A Smad Action Turnover Switch Operated by WW Domain Readers of a Phosphoserine Code. Genes Dev..

[182] Kamato D., Do B.H., Osman N., Ross B.P., Mohamed R., Xu S., Little P.J. (2019). Smad Linker Region Phosphorylation Is a Signalling Pathway in Its Own Right and Not Only a Modulator of Canonical TGF-β Signalling. Cell. Mol. Life Sci. CMLS.

[183] Wang G., Matsuura I., He D., Liu F. (2009). Transforming Growth Factor-β-Inducible Phosphorylation of Smad3. J. Biol. Chem..

[184] Gao S., Alarcón C., Sapkota G., Rahman S., Chen P.-Y., Goerner N., Macias M.J., Erdjument-Bromage H., Tempst P., Massagué J. (2009). Ubiquitin Ligase Nedd4L Targets Activated Smad2/3 to Limit TGF Bsignaling. Mol. Cell.

[185] Ooshima A., Park J., Kim S. (2019). Phosphorylation Status at Smad3 Linker Region Modulates Transforming Growth Factor-β-induced Epithelial-mesenchymal Transition and Cancer Progression. Cancer Sci..

[186] Firestein R., Bass A.J., Kim S.Y., Dunn I.F., Silver S.J., Guney I., Freed E., Ligon A.H., Vena N., Ogino S. (2008). CDK8 Is a Colorectal Cancer Oncogene That Regulates Beta-Catenin Activity. Nature.

[187] Firestein R., Hahn W.C. (2009). Revving the Throttle on an Oncogene: CDK8 Takes the Driver Seat. Cancer Res..

[188] Ahmad K., Brahma S., Henikoff S. (2024). Epigenetic Pioneering by SWI/SNF Family Remodelers. Mol. Cell.

[189] Cao G., Ma L., Dai X., Hou P., Yao X., Li G., Zhang J., Chen C., Lin W. (2025). ARID1A Governs Genomic Stability and Proliferation in SCLC via C-MYC/PARP1 Suppression Driving Vulnerability to BET Inhibitors. Research.

[190] Audetat K.A., Galbraith M.D., Odell A.T., Lee T., Pandey A., Espinosa J.M., Dowell R.D., Taatjes D.J. (2017). A Kinase-Independent Role for Cyclin-Dependent Kinase 19 in P53 Response. Mol. Cell. Biol..

[191] Menzl I., Zhang T., Berger-Becvar A., Grausenburger R., Heller G., Prchal-Murphy M., Edlinger L., Knab V.M., Uras I.Z., Grundschober E. (2019). A Kinase-Independent Role for CDK8 in BCR-ABL1+ Leukemia. Nat. Commun..

[192] Knab V.M., Gotthardt D., Klein K., Grausenburger R., Heller G., Menzl I., Prinz D., Trifinopoulos J., List J., Fux D. (2021). Triple-Negative Breast Cancer Cells Rely on Kinase-Independent Functions of CDK8 to Evade NK-Cell-Mediated Tumor Surveillance. Cell Death Dis..

[193] Li J., Hilimire T.A., Liu Y., Wang L., Liang J., Gyorffy B., Sikirzhytski V., Ji H., Zhang L., Cheng C. (2023). CDK19 and CDK8 Mediator Kinases Drive Androgen-Independent in vivo Growth of Castration-Resistant Prostate Cancer. bioRxiv.

[194] Brägelmann J., Klümper N., Offermann A., Von Mässenhausen A., Böhm D., Deng M., Queisser A., Sanders C., Syring I., Merseburger A.S. (2017). Pan-Cancer Analysis of the Mediator Complex Transcriptome Identifies CDK19 and CDK8 as Therapeutic Targets in Advanced Prostate Cancer. Clin. Cancer Res..

[195] Roninson I.B., Győrffy B., Mack Z.T., Shtil A.A., Shtutman M.S., Chen M., Broude E.V. (2019). Identifying Cancers Impacted by CDK8/19. Cells.

[196] Guzman J., Hart M., Weigelt K., Neumann A., Aigner A., Andolfi C., Handle F., Rheinheimer S., Fischer U., Immel U.D. (2025). The MicroRNA miR-454 and the Mediator Complex Component MED12 Are Regulators of the Androgen Receptor Pathway in Prostate Cancer. Sci. Rep..

[197] Taylor R.A., Fraser M., Livingstone J., Espiritu S.M.G., Thorne H., Huang V., Lo W., Shiah Y.-J., Yamaguchi T.N., Sliwinski A. (2017). Germline BRCA2 Mutations Drive Prostate Cancers with Distinct Evolutionary Trajectories. Nat. Commun..

[198] Grieb B.C., Eischen C.M. (2022). MTBP and MYC: A Dynamic Duo in Proliferation, Cancer, and Aging. Biology.

[199] Odvody J., Vincent T., Arrate M.P., Grieb B., Wang S., Garriga J., Lozano G., Iwakuma T., Haines D.S., Eischen C.M. (2010). A Deficiency in Mdm2 Binding Protein Inhibits Myc-Induced B-Cell Proliferation and Lymphomagenesis. Oncogene.

[200] Khatiwada P., Rimal U., Han Z., Shemshedini L. (2024). MDM2 Regulates the Stability of AR, AR-V7, and TM4SF3 Proteins in Prostate Cancer. Endocr. Oncol..

[201] Russo J.W., Nouri M., Balk S. (2019). Androgen Receptor Interaction with Mediator Complex Is Enhanced in Castration-Resistant Prostate Cancer by CDK7 Phosphorylation of MED1. Cancer Discov..

[202] ur Rasool R., Natesan R., Deng Q., Aras S., Lal P., Sander Effron S., Mitchell-Velasquez E., Posimo J.M., Carskadon S., Baca S.C. (2019). CDK7 Inhibition Suppresses Castration-Resistant Prostate Cancer through MED1 Inactivation. Cancer Discov..

[203] Jin F., Claessens F., Fondell J.D. (2012). Regulation of Androgen Receptor-Dependent Transcription by Coactivator MED1 Is Mediated through a Newly Discovered Noncanonical Binding Motif. J. Biol. Chem..

[204] Xiao L., Parolia A., Qiao Y., Bawa P., Eyunni S., Mannan R., Carson S.E., Chang Y., Wang X., Zhang Y. (2022). Targeting SWI/SNF ATPases in Enhancer-Addicted Prostate Cancer. Nature.

[205] Thienger P., Rubin P.D., Yao X., Benjak A., Shah S.R., Leung A.K.-Y., de Brot S., Naveed A., Shi M., Tremblay J. (2024). Androgen Receptor-Negative Prostate Cancer Is Vulnerable to SWI/SNF-Targeting Degrader Molecules. bioRxiv.

[206] Sandoval G.J., Pulice J.L., Pakula H., Schenone M., Takeda D.Y., Pop M., Boulay G., Williamson K.E., McBride M.J., Pan J. (2018). Binding of TMPRSS2-ERG to BAF Chromatin Remodeling Complexes Mediates Prostate Oncogenesis. Mol. Cell.

[207] Currie S.L., Doane J.J., Evans K.S., Bhachech N., Madison B.J., Lau D.K.W., McIntosh L.P., Skalicky J.J., Clark K.A., Graves B.J. (2017). ETV4 and AP1 Transcription Factors Form Multivalent Interactions with Three Sites on the MED25 Activator-Interacting Domain. J. Mol. Biol..

[208] Krasnyi A.M., Ivanov R.A., Minskaia E. (2026). Spatial and Epigenetic Polarity of the AR Promoter During Prostate Cancer Progression: An In Silico Analysis. Int. J. Mol. Sci..

[209] Jones T., Feng J., Luyties O., Cozzolino K., Sanford L., Rimel J.K., Ebmeier C.C., Shelby G.S., Watts L.P., Rodino J. (2025). TFIIH Kinase CDK7 Drives Cell Proliferation through a Common Core Transcription Factor Network. Sci. Adv..

[210] Vander Ark A., Cao J., Li X. (2018). Mechanisms and Approaches for Overcoming Enzalutamide Resistance in Prostate Cancer. Front. Oncol..

[211] Offermann A., Joerg V., Becker F., Roesch M.C., Kang D., Lemster A.-L., Tharun L., Behrends J., Merseburger A.S., Culig Z. (2022). Inhibition of Cyclin-Dependent Kinase 8/Cyclin-Dependent Kinase 19 Suppresses Its Pro-Oncogenic Effects in Prostate Cancer. Am. J. Pathol..

[212] Duong T., Araujo Rincon M., Myneni N., Burleson M. (2023). Genetic Alterations in MED12 Promote Castration-Resistant Prostate Cancer through Modulation of GLI3 Signaling. Mol. Biol. Res. Commun..

[213] Nakamura A., Nakata D., Kakoi Y., Kunitomo M., Murai S., Ebara S., Hata A., Hara T. (2018). CDK8/19 Inhibition Induces Premature G1/S Transition and ATR-Dependent Cell Death in Prostate Cancer Cells. Oncotarget.

[214] Constantin T.A., Greenland K.K., Varela-Carver A., Bevan C.L. (2022). Transcription Associated Cyclin-Dependent Kinases as Therapeutic Targets for Prostate Cancer. Oncogene.

[215] Richters A., Doyle S.K., Freeman D.B., Lee C., Leifer B.S., Jagannathan S., Kabinger F., Koren J.V., Struntz N.B., Urgiles J. (2021). Modulating Androgen Receptor-Driven Transcription in Prostate Cancer with Selective CDK9 Inhibitors. Cell Chem. Biol..

[216] Treatment—EAU Guidelines on Prostate Cancer—Uroweb. https://uroweb.org/guidelines/prostate-cancer/chapter/treatment.

[217] Fizazi K., Tran N., Fein L., Matsubara N., Rodriguez-Antolin A., Alekseev B.Y., Özgüroğlu M., Ye D., Feyerabend S., Protheroe A. (2017). Abiraterone plus Prednisone in Metastatic, Castration-Sensitive Prostate Cancer. N. Engl. J. Med..

[218] Davis I.D., Martin A.J., Stockler M.R., Begbie S., Chi K.N., Chowdhury S., Coskinas X., Frydenberg M., Hague W.E., Horvath L.G. (2019). Enzalutamide with Standard First-Line Therapy in Metastatic Prostate Cancer. N. Engl. J. Med..

[219] Chi K.N., Agarwal N., Bjartell A., Chung B.H., Pereira De Santana Gomes A.J., Given R., Juárez Soto Á., Merseburger A.S., Özgüroğlu M., Uemura H. (2019). Apalutamide for Metastatic, Castration-Sensitive Prostate Cancer. N. Engl. J. Med..

[220] Smith M.R., Hussain M., Saad F., Fizazi K., Sternberg C.N., Crawford E.D., Kopyltsov E., Park C.H., Alekseev B., Montesa-Pino Á. (2022). Darolutamide and Survival in Metastatic, Hormone-Sensitive Prostate Cancer. N. Engl. J. Med..

[221] Saad F., Vjaters E., Shore N., Olmos D., Xing N., Pereira De Santana Gomes A.J., Cesar De Andrade Mota A., Salman P., Jievaltas M., Ulys A. (2024). Darolutamide in Combination with Androgen-Deprivation Therapy in Patients with Metastatic Hormone-Sensitive Prostate Cancer From the Phase III ARANOTE Trial. J. Clin. Oncol..

[222] Ryan C.J., Smith M.R., Fizazi K., Saad F., Mulders P.F.A., Sternberg C.N., Miller K., Logothetis C.J., Shore N.D., Small E.J. (2015). Abiraterone Acetate plus Prednisone versus Placebo plus Prednisone in Chemotherapy-Naive Men with Metastatic Castration-Resistant Prostate Cancer (COU-AA-302): Final Overall Survival Analysis of a Randomised, Double-Blind, Placebo-Controlled Phase 3 Study. Lancet Oncol..

[223] Beer T.M., Armstrong A.J., Rathkopf D.E., Loriot Y., Sternberg C.N., Higano C.S., Iversen P., Bhattacharya S., Carles J., Chowdhury S. (2014). Enzalutamide in Metastatic Prostate Cancer before Chemotherapy. N. Engl. J. Med..

[224] Smith M.R., Saad F., Chowdhury S., Oudard S., Hadaschik B.A., Graff J.N., Olmos D., Mainwaring P.N., Lee J.Y., Uemura H. (2018). Apalutamide Treatment and Metastasis-Free Survival in Prostate Cancer. N. Engl. J. Med..

[225] Fizazi K., Shore N., Tammela T.L., Ulys A., Vjaters E., Polyakov S., Jievaltas M., Luz M., Alekseev B., Kuss I. (2019). Darolutamide in Nonmetastatic, Castration-Resistant Prostate Cancer. N. Engl. J. Med..

[226] De Bono J., Mateo J., Fizazi K., Saad F., Shore N., Sandhu S., Chi K.N., Sartor O., Agarwal N., Olmos D. (2020). Olaparib for Metastatic Castration-Resistant Prostate Cancer. N. Engl. J. Med..

[227] Sartor O., De Bono J., Chi K.N., Fizazi K., Herrmann K., Rahbar K., Tagawa S.T., Nordquist L.T., Vaishampayan N., El-Haddad G. (2021). Lutetium-177–PSMA-617 for Metastatic Castration-Resistant Prostate Cancer. N. Engl. J. Med..

[228] Aggarwal R.R., Vuky J., VanderWeele D., Rettig M., Heath E.I., Quigley D., Huang J., Chumber A., Cheung A., Foye A. (2025). Phase I, First-in-Human Study of FOR46 (FG-3246), an Immune-Modulating Antibody-Drug Conjugate Targeting CD46, in Patients with Metastatic Castration-Resistant Prostate Cancer. J. Clin. Oncol..

[229] Merck Sharp & Dohme LLC MK-2400-01A Substudy: A Phase 1/2, Open-Label Umbrella Substudy of MK-2400-U01 Master Protocol to Evaluate the Safety and Efficacy of Ifinatamab Deruxtecan-Based Treatment Combinations or Ifinatamab Deruxtecan Alone in Participants with Metastatic Castration-Resistant Prostate Cancer (mCRPC) (IDeate-Prostate02). https://clinicaltrials.gov/study/NCT06863272.

[230] Kyntra Bio A Phase 2 Dose Optimization Trial Evaluating a CD46-Targeted Antibody-Drug Conjugate (FG-3246) in Patients with Metastatic Castration-Resistant Prostate Cancer. https://clinicaltrials.gov/study/NCT06842498.

[231] BioNTech SE A Phase III, Randomized, Open-Label Trial of BNT324 Versus Docetaxel with Prednisone/Prednisolone in Metastatic Castration-Resistant Prostate Cancer. https://clinicaltrials.gov/study/NCT07365995.

[232] Bakht M.K., Beltran H. (2025). Biological Determinants of PSMA Expression, Regulation and Heterogeneity in Prostate Cancer. Nat. Rev. Urol..

[233] Emmett L., Swiha M., Papa N., Subramaniam S., Crumbaker M., Joshua A.M., Nguyen A., Weickhardt A., Lee S.-T., Ng S. (2026). Predictive Value of Early PSMA Upregulation for the Response to Enzalutamide ± 177Lu-PSMA-617 in Poor-Risk, Metastatic, Castration-Resistant Prostate Cancer: Substudy of the Randomized, Phase 2 ENZA-p Trial. Nat. Cancer.

[234] Tagawa S.T., Sartor O., Piulats J.M., Saad F., Fizazi K., Reid A., Beltran H., Kramer G., Mahammedi H., Eiber M. (2026). [177Lu]Lu-PSMA-617 in Patients with PSMA-Positive Metastatic Androgen Pathway Modulator-Naive/Sensitive Prostate Cancer (PSMAddition): A Phase 3 Randomised, Controlled Trial. Lancet.

[235] U.S. Food and Drug Administration FDA Approves Lutetium Lu 177 Vipivotide Tetraxetan with Androgen Receptor Pathway Inhibitor Therapy for Metastatic Androgen Pathway Modulation-Naïve or -Sensitive Prostate Cancer. https://www.fda.gov/drugs/resources-information-approved-drugs/fda-approves-lutetium-lu-177-vipivotide-tetraxetan-androgen-receptor-pathway-inhibitor-therapy.

[236] Xu K., Wu Z.J., Groner A.C., He H.H., Cai C., Lis R.T., Wu X., Stack E.C., Loda M., Liu T. (2012). EZH2 Oncogenic Activity in Castration-Resistant Prostate Cancer Cells Is Polycomb-Independent. Science.

[237] Pfizer A Phase 3, Randomized, Double Blind, Placebo Controlled Study of PF-06821497 (Mevrometostat) with Enzalutamide in Metastatic Castration Resistant Prostate Cancer (MEVPRO-2). https://clinicaltrials.gov/study/NCT06629779.

[238] Drescher C., Walter H.S., Gastinne T., Lakhani N.J., Ribrag V., Rasco D.W., Gutierrez M., Sullivan R.J., Harvey R.D., Banda K. (2023). EZH2/EZH1 Inhibitor Tulmimetostat (CPI-0209) in Patients with Advanced Solid Tumors or Hematologic Malignancies: Preliminary Phase II Results. J. Clin. Oncol..

[239] Novartis Pharmaceuticals A Phase 1/2 Study of DZR123 (CPI-0209) in Patients with Advanced Solid Tumors and Lymphomas. https://clinicaltrials.gov/study/NCT04104776.

[240] Biocad A Multicenter Open-Label Non-Comparative 2-Stage Phase 1a/1b Study to Assess the Safety and Pharmacokinetics of BCD-115 (JSC BIOCAD, Russia) in Combination with Endocrine Therapy in Women with ER(+) HER2(−) Local Advanced and Metastatic Breast Cancer. https://clinicaltrials.gov/study/NCT03065010.

[241] Ryvu Therapeutics SA An Open-Label, Single Agent, Phase I/II Trial Investigating the Safety and Efficacy of RVU120 (SEL120) in Patients with Relapse/Refractory Metastatic or Advanced Solid Tumors. https://clinicaltrials.gov/study/NCT05052255.

[242] Dziadziuszko R., Lugowska I., Garralda E., Rojas Laimito K.I., Rzymski T., Dudziak R., Littlewood P., Nogai H., Boni V., Fuentes Antras J. (2023). 672P Phase I/II Trial of RVU120 (SEL120), CDK8/CDK19 Inhibitor in Patients with Relapsed/Refractory Metastatic or Advanced Solid Tumors. Ann. Oncol..

[243] Tyligand Bioscience (Shanghai) Limited A Multicenter, Open-Label, Phase Ia/Ib Study to Evaluate the Safety, Tolerability, Pharmacokinetics, Pharmacodynamics, and Preliminary Efficacy of TSN084 Tablets in Patients with Advanced Malignant Tumors. https://clinicaltrials.gov/study/NCT06386705.

[244] Ho T.-Y., Sung T.-Y., Pan S.-L., Huang W.-J., Hsu K.-C., Hsu J.-Y., Lin T.E., Hsu C.-M., Yang C.-R. (2023). The Study of a Novel CDK8 Inhibitor E966-0530–45418 That Inhibits Prostate Cancer Metastasis in vitro and in vivo. Biomed. Pharmacother..

[245] Beltran H., Hruszkewycz A., Scher H.I., Hildesheim J., Isaacs J., Yu E.Y., Kelly K., Lin D., Dicker A., Arnold J. (2019). The Role of Lineage Plasticity in Prostate Cancer Therapy Resistance. Clin. Cancer Res..

[246] Aggarwal R., Huang J., Alumkal J.J., Zhang L., Feng F.Y., Thomas G.V., Weinstein A.S., Friedl V., Zhang C., Witte O.N. (2018). Clinical and Genomic Characterization of Treatment-Emergent Small-Cell Neuroendocrine Prostate Cancer: A Multi-Institutional Prospective Study. J. Clin. Oncol..

[247] Su Y., Liu Y., Behrens C.R., Bidlingmaier S., Lee N.-K., Aggarwal R., Sherbenou D.W., Burlingame A.L., Hann B.C., Simko J.P. (2018). Targeting CD46 for Both Adenocarcinoma and Neuroendocrine Prostate Cancer. JCI Insight.

[248] Shi X., Day A., Bergom H.E., Tape S., Baca S.C., Sychev Z.E., Larson G., Bozicevich A., Drake J.M., Zorko N. (2022). Integrative Molecular Analyses Define Correlates of High B7-H3 Expression in Metastatic Castrate-Resistant Prostate Cancer. npj Precis. Oncol..

[249] Guo C., Figueiredo I., Gurel B., Neeb A., Seed G., Crespo M., Carreira S., Rekowski J., Buroni L., Welti J. (2023). B7-H3 as a Therapeutic Target in Advanced Prostate Cancer. Eur. Urol..

[250] Clark A.D., Oldenbroek M., Boyer T.G. (2015). Mediator Kinase Module and Human Tumorigenesis. Crit. Rev. Biochem. Mol. Biol..

[251] Liu Y., Sun L., Lu H. (2025). A Comprehensive Description of Cyclin-Dependent Kinase 8 (CDK8) Inhibitors as Anticancer Agents. Bioorg. Med. Chem..

[252] Chen M., Li J., Liang J., Thompson Z.S., Kathrein K., Broude E.V., Roninson I.B. (2019). Systemic Toxicity Reported for CDK8/19 Inhibitors CCT251921 and MSC2530818 Is Not Due to Target Inhibition. Cells.

